# Most of the Specific Genomic Regions of *Agrobacterium fabrum* Shape Phenolics Content of *Medicago truncatula* Roots

**DOI:** 10.1111/1758-2229.70394

**Published:** 2026-08-07

**Authors:** Rosa Padilla, Ludovic Vial, Florence Dechèvre, Thi Huyen Thu Nguyen, Deniz Sarigol, Vincent Gaillard, Guillaume Meiffren, Gilles Comte, Xavier Nesme, Céline Lavire, Isabelle Kerzaon

**Affiliations:** ^1^ Université Lyon 1, CNRS, INRAE, LEM, UMR 5557, UMR 1418 Villeurbanne France

**Keywords:** *Agrobacterium*, *Medicago*, metabolomic, phenolic compounds, plant‐bacteria interaction, root metabolites, specific genes

## Abstract

The impact of plant microbiota on host is increasingly recognized, yet specific bacterial functions and genetic determinants in rhizosphere interactions remain largely unknown. *Agrobacteria*, common soil and rhizospheric bacteria, establish root interactions. The species *Agrobacterium fabrum* harbour seven specific‐gene regions, whose annotation indicates a close connection with the plant. To evaluate the involvement of these specific regions in the plant‐bacteria interaction, deletion mutant strains of each specific region were inoculated on *Medicago truncatula* roots. Root metabolite profiles were compared by UHPLC‐UV/DAD‐ESI‐MS QTOF analyses, and the highlighted discriminating metabolites were annotated by tandem mass spectrometry. *A. fabrum* inoculation modulates the content of root phenolic compounds, in particular flavonoids. These root metabolite modulations appear to be linked to at least one of the *A. fabrum*‐specific genes, as almost all specific regions showed an influence on one or more of these metabolites. Furthermore, our results suggested a putative cross‐talk of the specific regions during the interaction of *A. fabrum* with *M. truncatula* , as all mutants except one induced similar modifications on flavonoids. These findings enhance our understanding of *A. fabrum*'s ecological niche construction, highlighting the importance of specific genes in the establishment of this fine‐tuned interaction.

## Graphical abstract

*Agrobacterium fabrum* harbour seven specific‐gene regions, whose annotation indicates a close connection with plant. To evaluate their involvement in plant‐bacteria interaction, deletion mutant strains of each specific region were inoculated onto *Medicago truncatula* roots. Root phenolics content was influenced by them. These specific regions would play a role in this interaction.

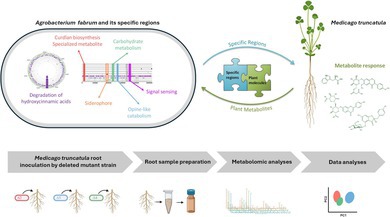

## 1 Introduction

The critical role and the diversity of the plant microbiota have gained widespread recognition. Microorganisms associated with the plant can profoundly impact their health: ranging from causing devastating diseases to promoting growth, health, and productivity. For certain well‐studied bacteria, such as *Xanthomonas* (a notorious plant pathogen) or nitrogen‐fixing rhizobia, we now have a solid understanding of their effects on plants (Philippot et al. 2013). For model bacteria or bacteria of economic interest, key genetic determinants driving these interactions have been identified, shedding light on the mechanisms underlying their influence. In the rhizosphere, however, the functions of most bacteria and the genetic determinants involved in the molecular dialogue between plants and bacteria are poorly understood.

Agrobacteria, a well‐known phytopathogenic bacteria, are responsible for crown gall disease affecting a wide range of plant species (Dessaux and Faure 2018). These bacteria are only pathogenic when they carry a plasmid named Ti, for tumour inducing (Hooykaas 2023). Agrobacteria are above all ubiquitous rhizospheric bacteria able to establish commensal or even beneficial interactions with plant roots (Costechareyre et al. 2010; Naqqash et al. 2016). Field investigations consistently showed that several species of agrobacteria co‐exist in the same biotopes (bare soil, rhizosphere, host plants) (Nesme et al. 1987; Vogel et al. 2003). This means that co‐existing species must exploit different resources to avoid competition with their closest relatives and thus have at least partly different ecological niches. A better understanding of these ecological niches and interactions with plants could provide insights for combating this pathogen. In this sense, *Agrobacterium* species harbour species‐specific genes (e.g., encoding specific ecological functions) potentially responsible for their adaptation to a species‐specific plant ecological niche (Lassalle et al. 2011, 2017).

Comparative genomics revealed that the species‐specific genes of the genomic species *Agrobacterium fabrum* (i.e., *Agrobacterium* species genomovar G8) were mainly clustered into seven genomic islands on its genome, called “specific‐regions” or SpG8 (for G8‐specific coding DNA sequences), going from SpG8‐1 to SpG8‐7 (Lassalle et al. 2011; Du et al. 2023). Some specific‐regions were subsequently divided into subclusters encoding homogeneous functions (Lassalle et al. 2011). Several specific‐regions encode coherent putative pathways related to the metabolism of trophic resources derived from plants (SpG8‐1a, SpG8‐1b, SpG8‐4, SpG8‐5), to the expression of environmental sensing systems (SpG8‐7), or to the synthesis of extracellular compounds, such as siderophores (SpG8‐3) or curdlan (SpG8‐2a) (Lassalle et al. 2011).

The latter, a glucose polymer induced under stress conditions, surrounds and protects bacteria against heat and desiccation (McIntosh et al. 2005; Matthysse 2018). Even if the role of curdlan in the attachment of *A. fabrum* to biotic or abiotic surfaces is not well documented, the genes involved in curdlan biosynthesis are upregulated when *A. fabrum* colonizes *Arabidopsis* (González‐Mula et al. 2018).

The SpG8‐1b encodes the complete degradation pathway of some hydroxycinnamic acids (HCA) that will ultimately be used as a source of carbon and energy by *A. fabrum* (Campillo et al. 2014; Meyer et al. 2018). HCA are common plant specialized metabolites released in soil during the decay of root cells and described as chemotactic signals for agrobacteria (Whitehead et al. 1983; Parke et al. 1987). This degradation pathway could lead to a competitive advantage of *A. fabrum* over other agrobacteria in HCA‐enriched environments such as the rhizosphere (Lassalle et al. 2011; Meyer et al. 2018).

The specific‐region SpG8‐3 encodes the biosynthesis of siderophores and reuptake/regulatory system required for *A. fabrum* C58 growth under iron limiting conditions (Rondon et al. 2004). These unusual siderophores, named fabrubactins A and B (Vinnik et al. 2021), could provide a fitness advantage to outperform competitors, especially in complex habitats like the rhizosphere (Lassalle et al. 2011). SpG8‐1b and SpG8‐3 specific‐regions have coordinated expression and are induced in the presence of HCAs (Baude et al. 2016). The combined expression of these two specific regions suggests that several of them could be involved in the adaptation to a plant‐related ecological niche (Lassalle et al. 2017).

The annotation of these specific regions leans towards a close connection with the plant. They are thus suspected to have an effect on root during bacteria‐plant interactions. The roots are highly colonized by a wide range of soil microorganisms, establishing different types of interactions which involve rhizosphere chemical dialogues via primary and specialized metabolites (Massalha et al. 2017). Indeed, plants can synthesize a wide diversity of specialized metabolites, which are known to be key components for the adaptation to abiotic and/or biotic conditions and notably the plant microbiome (Jan et al. 2021; Koprivova and Kopriva 2022). Metabolomic studies allow the identification of metabolites involved in plant‐bacteria interactions as it is a powerful approach to achieve an accurate comparative study of the metabolite content modulation (Gupta et al. 2022). Recent studies on the effect of phytobacteria on plants (Valette et al. 2019; Nong et al. 2023) highlighted significant changes in root metabolite profiles, in particular phenolic compounds. These compounds play multifunctional roles in plant‐microbial interactions, notably signalling molecules for rhizobacteria/agrobacteria (Mandal et al. 2010).

To evaluate the involvement of *A. fabrum*‐specific regions in the specific interaction with plant, investigations of their impact on the plant and specifically on its specialized metabolites were performed. To this end, deletion mutant strains of each *A. fabrum*‐specific region were inoculated on *Medicago truncatula* roots, a well‐established plant model frequently colonized by *Agrobacterium* (Cook 1999; Farag et al. 2008). Root metabolite profiles were analysed and compared by ultra–high‐performance liquid chromatography coupled to a diode array detector and an electrospray ionization quadrupole time‐of‐flight mass spectrometer (UHPLC‐UV/DAD‐ESI‐MS QToF). Highlighted discriminating metabolites were annotated/identified by tandem mass spectrometry (MS/MS). Almost all *A. fabrum‐*specific regions were found to influence specialized root metabolites (mainly flavonoid classes), in response to the interaction of *A. fabrum* with *M. truncatula* roots.

## 2 Material and Methods

### 2.1 Chemicals

Organic solvent for extraction was distilled methanol purchased from VWR Chemicals (Paris, France). Acetonitrile, water and acetic acid (Optima UHPLC‐grade, Fisher Scientific, Geel, Belgium) were used for UHPLC‐UV/DAD‐ESI MS QTOF analyses. Tryptophan was obtained from Agilent Technologies (Waidbronn, Germany). Flavonoids (i.e., 7,4′‐dihydroxyflavone, liquiritigenin, diosmetin, ononin, apigenin, maesopsin, formononetin, kaempferol and kaempferol 3‐O‐glucoside) were obtained from Extrasynthese (Genay, France). Maesopsin 4‐O‐glycoside was kindly provided by Pr. Laurence Voutquenne‐Nazabadioko from the Reims Institute of Molecular Chemistry (ICMR, UMR CNRS 7312).

### 2.2 Bacterial Strains, Plasmids and Culture Conditions

The bacteria and plasmids used for this study are listed in Table 1. *Escherichia coli* was grown with shaking (160 rpm) at 37°C in Lysogenic Broth (LB) medium. Growth media were supplemented with appropriate antibiotics (gentamicin 15 μg/mL, kanamycin 25 μg/mL) when necessary. The *A. fabrum* strains were grown at 28°C in Yeast Peptone Glucose (YPG)‐rich medium (yeast extract, 5 g⸱L^−1^; peptone, 10 g⸱L^−1^; glucose, 10 g⸱L^−1^; pH 7.2) liquid or solid when supplemented with agar (15 g⸱L^−1^) or Agrobacterium Broth (AB) minimal medium supplemented with succinate (10 mM) (Chilton et al. 1974). When necessary, growth media were supplemented with appropriate antibiotics (gentamicin 20 μg/mL, neomycin 25 μg/mL, kanamycin 25 μg/mL).

**TABLE 1:** Strains and plasmids used in this study.

Strains	Relevant genotype and features	References
*Escherichia coli*		
JM109	*endA1 glnV44 thi‐1 relA1 gyrA96 recA1 mcrB+ Δ(lac‐proAB) e14‐ [F'traD36 proAB+ lacIq lacZΔM15] hsdR17(rK‐mK+)*	NEB catalogue
*Agrobacterium fabrum*		
C58 (WT1)	Wild‐type	CFBP 1903
C58 Kan (WT2)	pTi*atu6148*:Km, Neo/Kan^R^	(Lang et al. 2013)
C58ΔSpG8‐1b (Δ1b)	C58 deleted of *atu1409 –atu1423* cluster; Neo/Kan^R^	(Lassalle et al. 2011)
C58ΔSpG8‐2a (Δ2a)	C58 deleted of *atu3054 – atu3059* cluster; Neo/Kan^R^	(Lassalle et al. 2011)
C58ΔSpG8‐2b (Δ2b)	C58 deleted of *atu3069 – atu3073* cluster; Neo/Kan^R^	Present work
C58ΔSpG8‐3 (Δ3)	C58 deleted of *atu3663 – atu3693* cluster; Neo/Kan^R^	(Baude et al. 2016)
C58ΔSpG8‐4 (Δ4)	C58 deleted of *atu3808 – atu3830* cluster; Neo/Kan^R^	Present work
C58ΔSpG8‐5 (Δ5)	C58 deleted of *atu3947 – atu3952* cluster; Neo/Kan^R^	Present work
C58ΔSpG8‐7a (Δ7a)	C58 deleted of *atu4285 – atu4294* cluster; Neo/Kan^R^	Present work
C58ΔSpG8‐7b (Δ7b)	C58 deleted of *atu4295 – atu4307* cluster; Neo/Kan^R^	Present work
Plasmids		
pGEM‐T‐Easy	Cloning vector for PCR fragments; Amp^R^; *lacZ*	Promega
pOT1e	Promoter‐probe vector based on pBBR1MCS‐5 replicon; contains promoterless eGFP and MCS between two transcriptional terminators; Gm^R^	(Allaway et al. 2001)
pJQ200sk	Suicide vector; P15A *sacB*; Gm^R^	(Quandt and Hynes 1993)
pGEM‐T P*atu1416*	Upstream region of *atu1416* gene cloned into pGEM‐T‐Easy; Amp^R^	Present work
pGEM‐T P*atu3057*	Upstream region of *atu3057* gene cloned into pGEM‐T‐Easy; Amp^R^	Present work
pGEM‐T P*atu3069*	Upstream region of *atu3069* gene cloned into pGEM‐T‐Easy; Amp^R^	Present work
pGEM‐T P*atu3675*	Upstream region of *atu3675* gene cloned into pGEM‐T‐Easy; Amp^R^	Present work
pGEM‐T P*atu3817*	Upstream region of *atu3817* gene cloned into pGEM‐T‐Easy; Amp^R^	Present work
pGEM‐T P*atu3948*	Upstream region of *atu3948* gene cloned into pGEM‐T‐Easy; Amp^R^	Present work
pGEM‐T P*atu4292*	Upstream region of *atu4292* gene cloned into pGEM‐T‐Easy; Amp^R^	Present work
pGEM‐T P*atu4299*	Upstream region of *atu4299* gene cloned into pGEM‐T‐Easy; Amp^R^	Present work
pOT1e P*atu1416*	Upstream region of *atu1416* gene cloned into pOT1e; Gm^R^	Present work
pOT1e P*atu3057*	Upstream region of *atu3057* gene cloned into pOT1e; Gm^R^	Present work
pOT1e P*atu3069*	Upstream region of *atu3069* gene cloned into pOT1e; Gm^R^	Present work
pOT1e P*atu3675*	Upstream region of *atu3675* gene cloned into pOT1e; Gm^R^	Present work
pOT1e P*atu3817*	Upstream region of *atu3817* gene cloned into pOT1e; Gm^R^	Present work
pOT1e P*atu3948*	Upstream region of *atu3948* gene cloned into pOT1e; Gm^R^	Present work
pOT1e P*atu4292*	Upstream region of *atu4292* gene cloned into pOT1e; Gm^R^	Present work
pOT1e P*atu4298*	Upstream region of *atu4298* gene cloned into pOT1e; Gm^R^	Present work

### 2.3 Construction of the Deletion Mutants and Transcriptional Fusions

*A. fabrum* deletion mutants were constructed by recombination with the suicide vector pJQ200sk according to a strategy previously described by Lassalle et al. (2011). Briefly, the recombinant region containing the flanking fragments of the sequence to be deleted (primers listed in Table S1) and a fragment encoding the *nptII* gene (neomycin/kanamycin resistance) amplified from plasmid KD4 (Datsenko and Wanner 2000) were cloned into pJQ200sk (Quandt and Hynes 1993) and then inserted into *A. fabrum* C58 by electroporation. Single‐crossover integration was selected by kanamycin and neomycin resistance on YPG medium plates. Double crossover events were identified on YPG medium plates containing 10% sucrose. Deletion mutants were verified by diagnostic PCR with appropriate primers (Table S1) and DNA sequencing (GenoScreen, Lille, France).

The eGFP (enhanced Green Fluorescent Protein) transcriptional fusions were obtained as follows. The promoter regions of the targeted genes in each *A. fabrum* specific region were PCR amplified with specific primers listed in Table S1. Then, PCR fragments obtained were cloned into the pGEM‐T Easy vector (Promega, Madison, WI) according to manufacturer's instructions. After digestion of the resulting plasmids with HindIII and SalI, fragments were subcloned into pOT1e (Allaway et al. 2001) digested with the same enzymes. Finally, transcriptional reporter constructions were inserted into C58 by electroporation, and gentamicin resistant colonies were selected. Constructions were verified by diagnostic PCR using pOT1eF and pOT1eR primers listed in Table S1.

### 2.4 Phenotypic Characterization

C58 wild‐type (WT) and mutants for the specific‐regions were subjected to phenotypic characterization using Biolog phenotype MicroArrays PM1 and PM2A (Biolog Inc., Hayward, CA, United States). Briefly, all strains were grown on YPG medium for 48 h at 28°C and bacteria were collected from the agar surface and suspended in inoculating fluid (IF‐0) to a cell density of 42% transmittance on a turbidimeter (Biolog Inc., Hayward, CA, USA). 5 mL of cell suspension were added to 25 mL of IF‐0 + dye MixA to obtain a final transmittance of 85%. Each well of PM1 and PM2A plates was inoculated with 100 μL of this suspension. Plates were incubated at 28°C in the Omnilog for 60 h with readings taken every 20 min. After incubation, the Omnilog system was used to measure carbon substrate utilization. Biolog phenotypic MicroArray analyses were performed independently twice.

### 2.5 Plant Material and In Vitro Culture Procedures

All plant experiments were conducted on *Medicago truncatula* Gaertn (cultivar Jemalong line A17). *M. truncatula* seeds were surface disinfected by soaking in a 3.5% sodium hypochlorite solution before being thoroughly rinsed five times with sterile distilled water. To vernalize the seeds, they were put in the dark at 4°C overnight before use. The seeds were then scarified and placed in square Petri dishes (120 × 120 mm) containing 0.8% agar plant medium (A7921, Sigma Aldrich, St Louis, MO, USA) supplemented with nutrient solution (15% nitrogen, 15% potassium, 30% phosphorus at 1.5 g/L, Plant‐Prod 15‐15‐30, Fertil s.a.s., Le Syndicat, France). All Petri dishes were placed vertically, and the lower part was covered to protect the root development from light. The plants were incubated for 14 days in a phytotron with 16 h light daily and a temperature between 24°C and 28°C.

### 2.6 Confocal Microscopy Observation

For confocal observation, bacterial cells harbouring the eGFP transcriptional fusions were inoculated on the *M. truncatula* seeds with 10 μL of overnight culture (2 × 10^5^ bacteria⸱mL^−1^). At 14 days post‐inoculation (dpi), *M. truncatula* roots were mounted between a slide and a coverslip in a commercial mounting fluid (Aqua Poly/Mount, Polysciences Inc., Warrington, PA). Microscope observation was performed using a confocal laser scanning microscope (LSM 800 Meta Confocal Microscope, Zeiss, Oberkochen, Germany). eGFP was excited with an argon laser at 488 nm, and fluorescence was monitored at 528 nm. Analyses of images (five plants per condition) were performed with the LSM 800 software (Zeiss, Oberkochen, Germany).

### 2.7 Bacterial Root Colonization

Before inoculation with *A. fabrum*, scarified seeds were placed on plates containing 0.8% agar plant cell culture for 2 days in the darkness. Seedlings were inoculated with 5 μL of bacterial culture (prepared from overnight culture in YPG, washed with NaCl 0.8% and resuspended at 10^5^ CFU/mL in NaCl 0.8%) from *A. fabrum* WT and mutants. For each strain, concentration of the inoculum was verified by CFU (Colony Forming Colony) determination on YPG agar medium. Petri dishes with plants were placed vertically and the lower part was covered to protect the root development from light. The plants were incubated for 14 days in a phytotron with 16 h light daily and day/night temperature 20°C/18°C. To determine bacterial colonization level, roots were ground at 14 dpi. Serial dilutions were plated on YPG medium; colonies were counted after 2 days of incubation at 28°C. Bacteria abundance was expressed as log10 CFU per gram of root dry weight.

### 2.8 Bacterial Growth Assays With Plant Compounds

Bacterial strains were grown overnight in 15 mL of YPG medium at 28°C. Overnight cultures were centrifuged (7000 × g; 10 min), washed in NaCl 0.8%, and resuspended in YPG to obtain OD_600nm_ = 0.05. After a 4‐h period at 28°C under agitation, the cells were washed in NaCl 0.8% and diluted in 200 μL of AB succinate medium (10 mM) to an OD_600nm_ = 0.025. This AB medium was supplemented with either L‐tryptophan, liquiritigenin, kaempferol, ononin, diosmetin, maesopsin, maesopsin 4‐O‐glycoside (for C58 WT only), formononetin, or 7,4′‐dihydroxyflavone at 500 μM or apigenin at 200 μM. Control conditions were inoculated with sterile dH_2_O. Bacterial growth (OD_600nm_) was monitored in triplicate in Bioscreen plates using a Bioscreen plate reader (96 h, 28°C, shaking every 20 min before measurement). The experiment was done in two biological replicates. Bacterial growth data were analysed using R software (v4.5.1) with a linear mixed‐effects modelling (LMM) approach to account for the longitudinal nature of the data. To capture non‐linear growth kinetics, time was modelled using natural cubic splines (df = 4). The plots were created using the ggplot2 package (v4.0.0). At the end of the experiment, the contents of each well (with and without bacteria as control) were analysed by UHPLC‐UV/DAD‐ESI‐MS QTOF to assess the degradation of the compounds by the bacteria (see detail in section Metabolites analysis by UHPLC‐UV/DAD‐ESI‐MS QTOF).

### 2.9 Metabolomic Study of Plant‐Bacteria Interaction

#### 2.9.1 Plant Bacteria Co‐Culture Procedures

To perform metabolomic study, *Agrobacterium* strains were inoculated directly into the agar plant medium prior to seed deposition (at a concentration of 1 × 10^7^ bacteria⸱mL^−1^). One group of *M. truncatula* seeds was cultivated without bacterial inoculation to carry out the non‐inoculated (NI) condition as a control. All petri dishes were placed vertically, and the lower part was covered to protect the root development from light. The plants were incubated for 14 days in a phytotron with 16 h light daily and a temperature between 24°C and 28°C.

#### 2.9.2 Roots Harvesting and Extraction of Metabolites

After 14 days of incubation, the roots were harvested, pooled, frozen in liquid nitrogen (metabolism quenching), and freeze‐dried. They were then grinded using a bead mill TissueLyser II system (Qiagen, Hilden, Germany). Four or five replicates were performed per condition, each replicate consisting of root pools of several seedlings (between 7 and 12 to have enough and similar root dry mass for analyses). Dried powders were first extracted twice with methanol 80% and then extracted twice with pure methanol with homogenization and 10 min of sonication at room temperature. The combined extracts were then centrifuged (10 min, 19,745 × g), and the supernatants, particle free, were evaporated to dryness at 30°C in a Centrivap concentrator (Labconco, USA) to constitute the dried crude extracts. These extracts were weighed, dissolved in methanol 60% at 10 mg/mL, and conserved at −80°C prior to analysis. A quality control (QC) sample was prepared containing 20 μL of each extract. A bacterial extract was also prepared following a similar extraction process applied to bacterial culture performed without plant, which constitutes another type of control.

#### 2.9.3 Metabolites Analysis by UHPLC‐UV/DAD‐ESI‐MS QTOF

Metabolites analysis was performed on an UHPLC Agilent 1290 coupled to a UV–vis Diode Array Detector (Agilent 1290 Infinity series) and an Accurate‐Mass Q‐ToF 6530 spectrometer (Agilent Technologies). Liquid chromatography was carried out using a Nucleodur Sphinx RP C_18_ column (2 × 100 mm, 1.8 μm, Macherey‐Nagel) maintained at 50°C with an injection volume of 3 μL of sample. The mobile phase was a mixture of acetonitrile and acidified UHPLC‐grade water (0.4% acetic acid) applied at a flow rate of 0.5 mL/min, and with the following gradient (acidified H_2_O:CH_3_CN, [v/v]) starting at 98:2 for 1.5 min, increasing to 76:24 at 24.5 min, going to 0:100 in 4 min and maintained for 2 min, before returning to the starting conditions in 1 min and equilibrating for 2 min. The QTOF‐MS instrument was operated for MS analyses under the following conditions: the ion source ESI (Agilent Jet Stream thermal gradient focusing Technology) in positive ionization mode, with a 320°C gas temperature, an 11 L/min gas flow, a nebulizer pressure at 40 psi, a 360°C sheath gas temperature with 12 L/min flow rate, and with the capillary, nozzle, and fragmentor voltages at 3000, 500, and 150 V, respectively. The acquisition mass range was from *m/z* 100 to 2000. The QC sample was initially analysed and then injected after every 8–9 samples in the run sequence to monitor the repeatability of the analysis. For characterization of discriminating compounds, complementary MS and tandem MS experiments were performed in positive and negative ionization modes and with different collision energies (10, 20, 30 or 40 V) in AutoMS/MS mode. The UHPLC‐UV/DAD‐ESI‐MS QTOF device was managed by the MassHunter Workstation Acquisition B.07.00 software and the resulting data was reprocessed with the MassHunter Qualitative Analysis B.07.00 software (Agilent Technologies). Identification or annotation of discriminating metabolites were performed by the analysis of the UV–vis spectrum and the positive and negative MS and MS/MS spectra. These data were compared to in‐house database (SeMetI), and to literature data, first on compounds already described in *M. truncatula* but also on chemical data of metabolites from other plants. When possible, comparisons with standards (the entire molecule or the aglycone moiety) allowed confirming metabolite identity or at least the aglycone nature. Based on their accurate mass, all the discriminating plant metabolites were searched in the control bacterial extracts. None of these metabolites were detected (data not shown). Likewise, the bacterial metabolites found in the bacterial extract were not detected in the obtained root plant extracts.

#### 2.9.4 Metabolite Profiling Data Treatment and Statistical Analyses

First, integration of each peak of the UV‐chromatograms at 280 nm (wavelength allowing the detection of a wide range of specialized metabolites and especially phenolic compounds) was performed. The retention time alignment between all samples was made for each peak, resulting in a metabolite matrix with samples in rows and absolute areas (*A*_abs_) of the 92 detected metabolites in columns. Then for each metabolite within a given sample, its relative peak area (i.e., relative abundance in the sample = *A*_rel_) was calculated over the total area of the sample chromatogram (Σ*A*_abs_) as follows: %*A*_rel_ = *A*_abs_/Σ*A*_abs_ × 100. In the resulting metabolite matrix, only the most abundant metabolites (relative abundance > 1%) were considered to continue the study (28 peaks). Partial least squares discriminating analysis (PLS‐DA) was made from the data matrix to visualize the global effect of bacterial inoculation (WT strain, mutant strains and the NI condition) on root phenolic profiles of *M. truncatula* . Principal Component Analyses (PCAs) were made between the WT strain condition and each mutant strain condition to more precisely stand out the effect of each *A. fabrum* specific‐regions on root specialized metabolite profiles. The statistical study was oriented to a *t*‐test to compare each condition with the WT strain condition to highlight significantly different metabolites. Statistical analyses were performed using R software (RStudio version 0.99.491).

## 3 Results

In order to evaluate the influence of specific regions on root metabolites during the *A. fabrum‐M. truncatula* interaction, mutant strains with precise deletion in the specific‐regions (i.e., specific genes of *A. fabrum* species) were obtained previously or in this study (Tables 1 and 2). All the genetic constructions were performed in the model strain *A. fabrum* C58.

**TABLE 2:** Characteristics and function annotations of *A. fabrum* gene specific regions (Lassalle et al. 2011).

G8 specific‐regions	C58 CDSs^a^	Main predicted functions
SpG8‐1b	Atu1409–Atu1423	Ferulic acid/HCA uptake and catabolism^b^
SpG8‐2a	Atu3054–Atu3059	Curdlan exopolysaccharide (EPS) biosynthesis^b^
SpG8‐2b	Atu3069–Atu3073	Secondary metabolite biosynthesis
SpG8‐3	Atu3663–Atu3691	Siderophore biosynthesis (fabrubactins A and B); iron‐siderophore uptake^b^
SpG8‐4	Atu3808–Atu3830	Ribose transport; monosaccharide catabolism and carbohydrate metabolism
SpG8‐5	Atu3947–Atu3952	Opine‐like compounds catabolism
SpG8‐7a	Atu4285–Atu4294	Environmental signal sensing/transduction
SpG8‐7b	Atu4295–Atu4307	Environmental signal sensing/transduction

^a^ Coding DNA sequences.

^**b**^ Predicted functions were confirmed by experimental validation (Rondon et al. 2004; Lassalle et al. 2011; Campillo et al. 2014; Vinnik et al. 2021).

### 3.1 Phenotypic Characterization and Root Colonization Capacity of Mutants in the Specific‐Regions

First, no significant difference in growth rate was observed in vitro (in rich medium and minimal medium) between WT C58 and the deletion mutant strains, or between mutant strains (data not shown). Biolog phenotype MicroArrays were used to further explore the functions of specific‐regions. All but three deletion mutant strains showed the same use of carbon sources as the WT strain. C58ΔSpG8‐3, C58ΔSpG8‐5, and C58ΔSpG8‐7a mutant strains are different from the WT strain in their use of several hexuronic acid substrates (Figure S1). The most important decrease was observed in the presence of gluconic acid for C58ΔSpG8‐3 and of galacturonic and glucuronic acids for C58ΔSpG8‐5.

In a second step, the non‐detrimental effect of these mutations on the root colonization was verified. WT C58 and all the deletion mutant strains of *A. fabrum* were singly inoculated on *M. truncatula* germinating seeds and their abundance on roots was determined at 14 dpi. Some similar levels of colonization were observed between the WT and the deletion mutant strains (Figure S2). Thus, these *A. fabrum*‐specific region deletions have not been detrimental for the establishment and survival of the mutant bacterial strains on the roots of *M. truncatula* seedlings in vitro‐cultivated.

### 3.2 All *A. fabrum* Specific Regions Are Expressed on *M. truncatula* Roots

The expression of all these specific‐regions on *M. truncatula* roots was also validated with eGFP transcriptional fusion. For each specific‐region, one promoter was selected based on annotation of genes and used for transcriptional fusion construction with eGFP (Table 1). Each transcriptional fusion was introduced in the WT C58 strain and inoculated on *M. truncatula* seedlings. All transcriptional fusions in the WT strains are expressed in contact with the *M. truncatula* root system (Figure S3). Almost one gene of all the *A. fabrum*‐specific regions was activated during its interaction with *M. truncatula* roots.

### 3.3 Effect of *A. fabrum* Wild‐Type or Mutant Strains Inoculation on *M. truncatula* Seedlings Biomass

**FIGURE 1:**
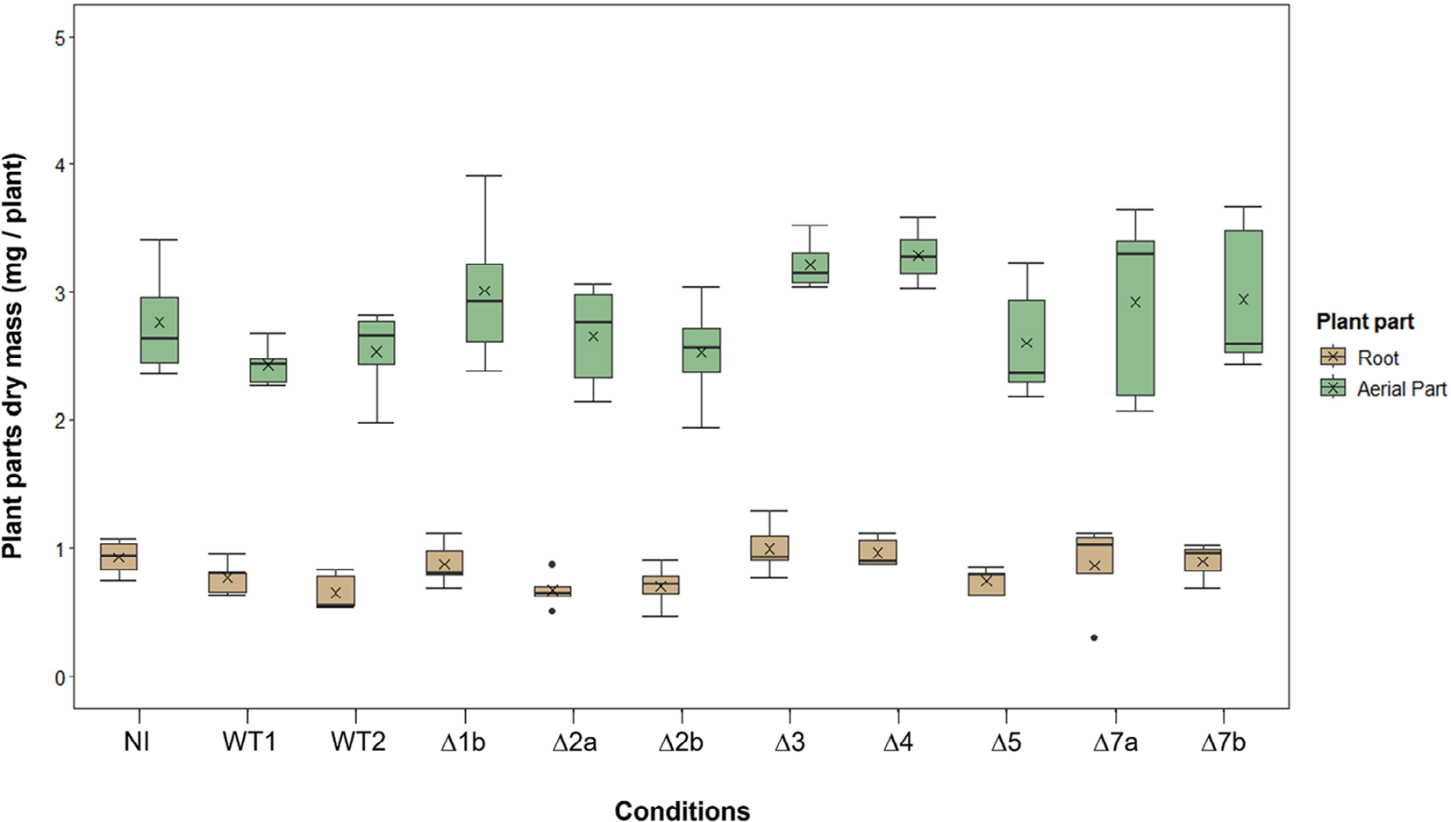
Boxplots illustrating the dry mass of roots and aerial parts from *M. truncatula* inoculated or not by *A. fabrum* wild‐type or deletion mutant strains. For each condition at 14 dpi, the roots and aerial parts of *M. truncatula* in vitro cultivated were harvested and dried. Dry masses of these plant parts were weighed and expressed as mg per seedling. Boxes cover 50% of the data. Central lines represent the medians and whiskers represent the minimum and maximum values among non‐atypical data. The cross (×) denotes the mean value of the data (*n* = 4 or 5). Similar dry masses were obtained on the one hand for roots (mean values ± SE, from 0.65 ± 0.04 to 1.00 ± 0.15 mg dry root per plant) and on the other for aerial parts (mean values ± SE, from 2.43 ± 0.07 to 3.29 ± 0.33 mg dry aerial part per plant), whether *M. truncatula* seedlings were non‐inoculated (NI) or inoculated with the wild‐type (WT1, WT2) or the deletion mutant strains (Δ1b = C58ΔSpG8‐1b; Δ2a = C58ΔSpG8‐2a; Δ2b = C58ΔSpG8‐2b; Δ3 = C58ΔSpG8‐3, Δ4 = C58ΔSpG8‐4; Δ5 = C58ΔSpG8‐5; Δ7a = C58ΔSpG8‐7a; Δ7b = C58ΔSpG8‐7b). No statistically significant difference was observed for each plant part (Dunn's test pairwise comparisons using *p*‐values adjusted with the Bonferroni method, for roots of all conditions *p* > 0.322, for aerial parts of all conditions *p* > 0.285).

All these *A. fabrum* strains (WT C58 and deletion mutant strains) were individually inoculated onto the root of *M. truncatula* germinating seeds to evaluate their impact on the plant biomass compared to uninoculated plants. After 14 days of in vitro co‐culture, *M. truncatula* roots and aerial parts were separately harvested and dried. Whether *M. truncatula* seedlings were non‐inoculated or inoculated (regardless of strain), similar dry matters were obtained from roots and from aerial parts (Figure 1). At 14 dpi under our conditions, inoculation of seedlings with *A. fabrum* strains (WT C58 or deletion mutants) did not affect *M. truncatula* development in terms of root and aerial part dry matter.

### 3.4 Influence of *A. fabrum* Inoculation on Root Specialized Metabolites of *M. truncatula*

At 14 dpi, the harvested and dried roots of *M. truncatula* were extracted with methanol. Whether *M. truncatula* seedlings were non‐inoculated or inoculated with the WT C58 strain or the deletion mutant strains, similar amounts of crude extract were obtained from roots (Figure S4).

**FIGURE 2:**
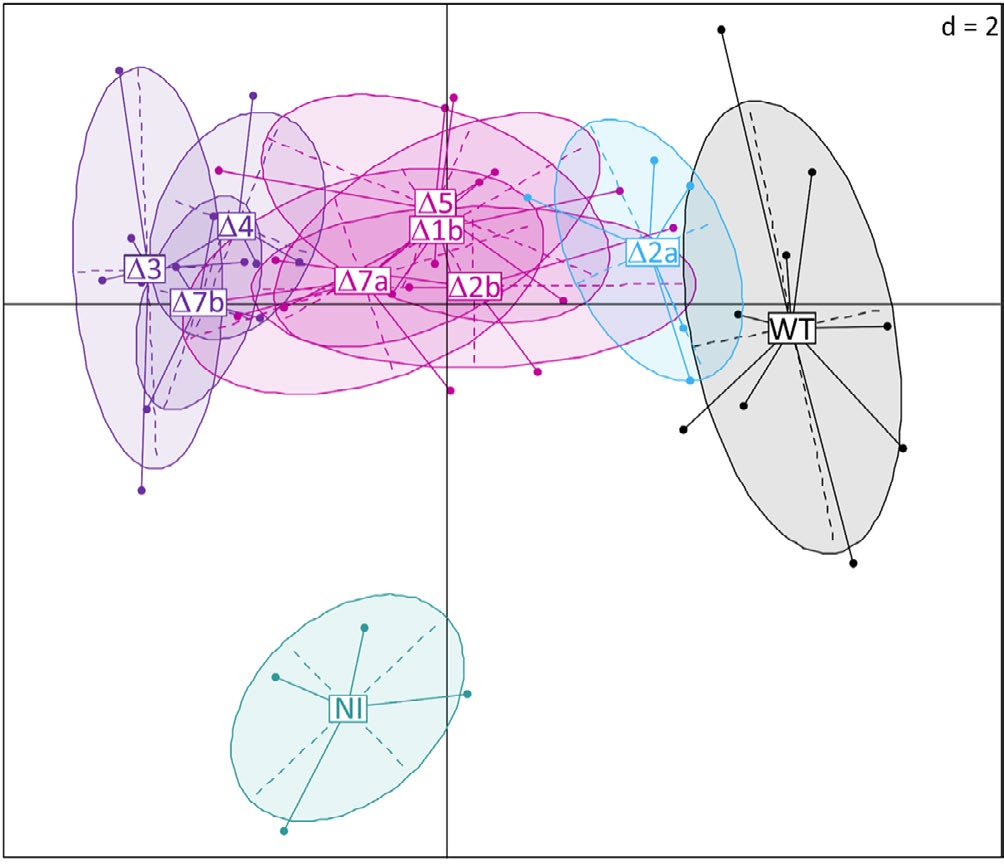
PLS‐DA score plot of root specialized metabolites profiles of *M. truncatula* seedlings non‐inoculated (NI) or inoculated with wild‐type (WT) or mutant (Δx) strains of *A. fabrum* C58. PLS‐DA were performed on the data matrix from chromatographic data (280 nm) obtained for each methanolic extract of *M. truncatula* roots based on peak areas and retention time (peaks > 1%). Plants were inoculated or not with *A. fabrum* WT C58 or deletion mutant strains of *A. fabrum*‐specific regions. WT: Plants inoculated with the wild‐type strain (in black), NI: Plants non‐inoculated (in cyan), Δx: Plants inoculated singly with each of the deletion mutant of *A. fabrum‐*specific regions (e.g., Δ2a corresponds to plants inoculated with the C58ΔSpG8‐2a mutant strain). In blue are drawn the closest root metabolite profiles to the one of WT condition, in purple the further ones, and in pink the intermediate profiles. Δ1b = C58ΔSpG8‐1b; Δ2a = C58ΔSpG8‐2a; Δ2b = C58ΔSpG8‐2b; Δ3 = C58ΔSpG8‐3; Δ4 = C58ΔSpG8‐4; Δ5 = C58ΔSpG8‐5; Δ7a = C58ΔSpG8‐7a; Δ7b = C58ΔSpG8‐7b.

**FIGURE 3:**
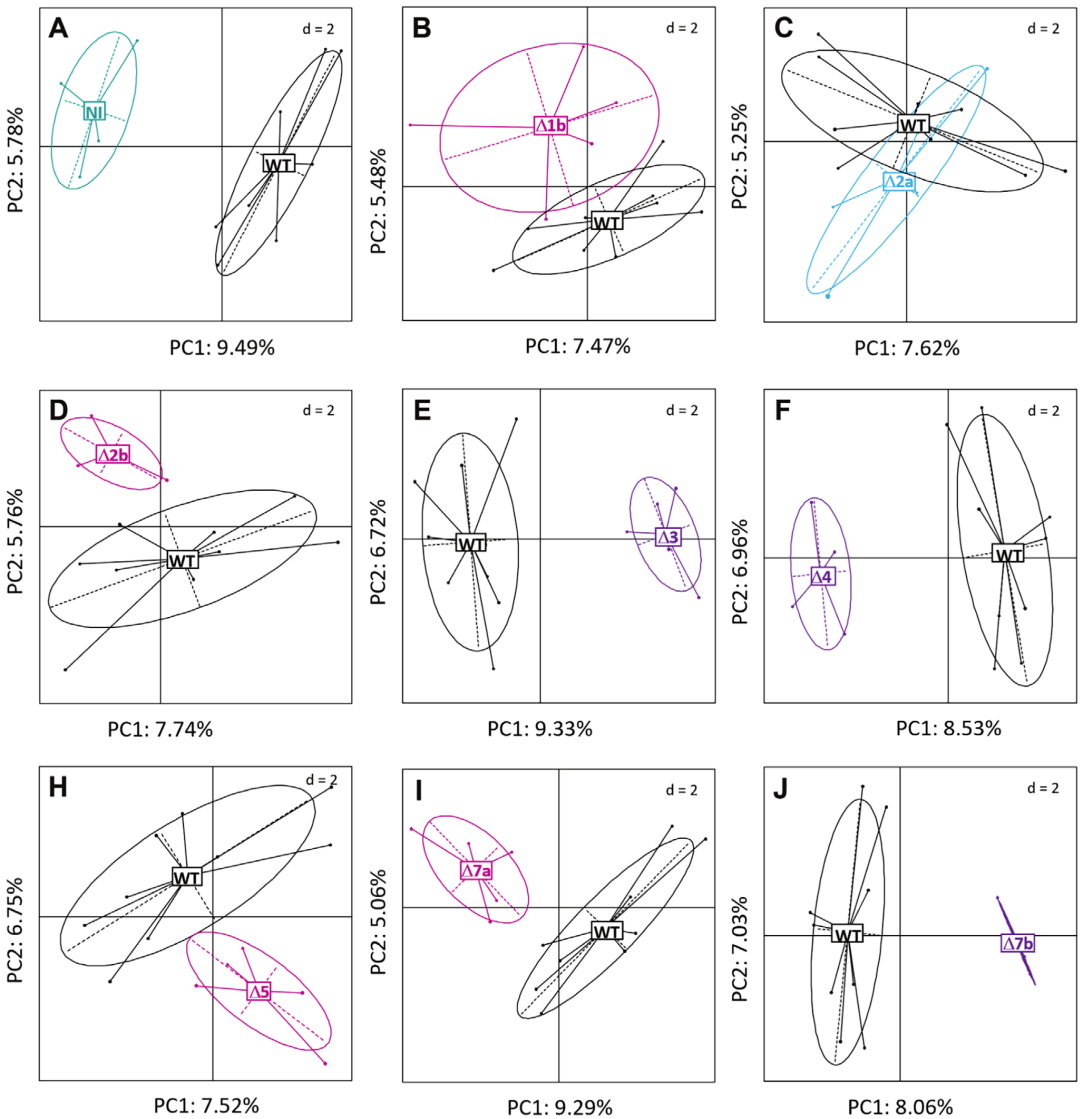
PCA score plots representing the comparison of root specialized metabolite profiles of *M. truncatula* seedlings between plants inoculated with wild‐type strain (WT) and plants non‐inoculated (NI) or inoculated with mutant (Δx) strains of *A. fabrum* C58. PCAs were performed on chromatographic data at 280 nm obtained for each methanolic extract of *M. truncatula* roots based on peak areas and retention time (peaks > 1%). Plants were inoculated or not with *A. fabrum* WT C58 or deletion mutant strains of *A. fabrum*‐specific regions. WT: Plants inoculated with the wild‐type strain (in black), NI: Plants non‐inoculated (in cyan), Δx: Plants inoculated singly with each of the deletion mutant of *A. fabrum‐*specific regions (e.g., Δ2a corresponds to plants inoculated with the C58ΔSpG8‐2a mutant strain). In blue the closest metabolite profiles to the one of WT are drawn, in purple the further ones, and in pink the intermediate profiles (colour similar to that used in Figure 2). Δ1b = C58ΔSpG8‐1b; Δ2a = C58ΔSpG8‐2a; Δ2b = C58ΔSpG8‐2b; Δ3 = C58ΔSpG8‐3; Δ4 = C58ΔSpG8‐4; Δ5 = C58ΔSpG8‐5; Δ7a = C58ΔSpG8‐7a; Δ7b = C58ΔSpG8‐7b.

The specialized metabolite profiles of *M. truncatula* roots between plants inoculated with wild‐type C58 strain (WT condition) and non‐inoculated plants (NI condition) or plants inoculated with deletion mutant strains of *A. fabrum* (mutant strain conditions) were compared. Root methanolic extracts were analysed to obtain chromatograms at 280 nm, a wavelength allowing the detection of a wide range of plant specialized metabolites and especially phenolic compounds. The quality control (QC) analysis led to the detection of 92 peaks (Figure S5). The variations in chromatographic profiles observed between conditions lie in the relative abundance of peaks rather than in the appearance or disappearance of peaks. Chromatographic profiles were visualized globally using PLS‐DA (Figure 2) and precisely compared using PCA (Figure 3).

Two conditions corresponding to *A. fabrum* WT strain were used in this study (named WT1 and WT2): one was a native *A. fabrum* wild‐type strain (WT1) and the second (WT2) had the *nptII* kanamycin resistance gene used to construct deletion mutants. A first PCA comparison showed no separation of *M. truncatula* root metabolite profiles between these two WT conditions (Figure S6). The statistical analyses on each peak highlighted only one (peak **39**) significantly different in its relative abundance between WT1 and WT2. As this peak was influenced by the presence of the *nptII* gene, it was excluded from the data matrix for further analysis. The two WT conditions were then combined into a single condition, henceforth referred to as the WT condition.

#### 3.4.1 Global Visualization of the Root Specialized Metabolite Profiles

The PLS‐DA performed on all root metabolite profiles provided a global visualization (Figure 2). On the one hand, the root metabolite profile of the NI condition seemed to be distinct from all the other profiles of the inoculated conditions. On the other hand, a gradient of root metabolite profiles seemed to appear between plants inoculated by the WT C58 strain and deletion mutants of *A. fabrum*‐specific regions strains (Figure 2). The root metabolite profile of plants inoculated with mutant strain C58ΔSpG8‐2a seemed to be the closest one to the WT condition, while that of plants inoculated with mutant strain C58ΔSpG8‐3 seemed to be furthest apart. However, as it describes a large number of conditions for a relatively small number of variables, this PLS‐DA representation cannot be considered as a predictive model.

#### 3.4.2 Evidence of Bacterial Effects on Root Specialized Metabolites Content

**FIGURE 4:**
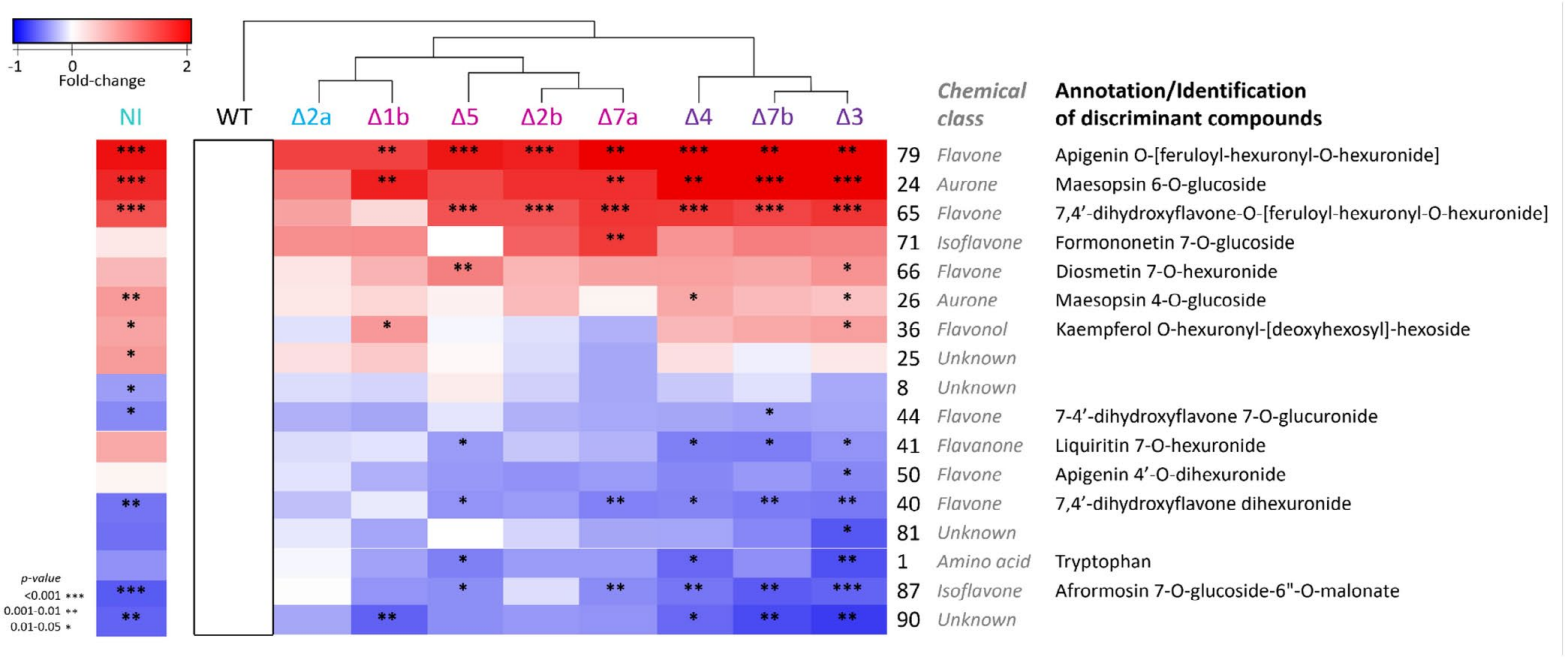
Heatmap of significantly discriminant metabolites according to their relative abundance in the roots of *Medicago truncatula* between plants inoculated with wild‐type strain (WT) and plants non‐inoculated (NI) or inoculated with mutant (Δx) strains of *A. fabrum* C58. Metabolites are represented as numbers on the right‐hand side of the plot, using the metabolite number obtained from the data processing. Chemical class and annotation/identification are indicated when determined. These compounds are over‐abundant (red) or under‐abundant (blue) in plants either not inoculated (NI) or inoculated with each of the deletion mutant strains of *A. fabrum*‐specific regions (Δx) compared to plants inoculated with C58 wild‐type (WT) strain of *A. fabrum* used as reference. The colours of the mutant strains are similar to those used in Figures 2 and 3. The *A. fabrum*‐specific regions deleted in the mutant strains Δx and their predicted functions are as follows: Δ1b = C58ΔSpG8‐1b (Ferulic acid/HCA uptake and catabolism); Δ2a = C58ΔSpG8‐2a (curdlan EPS biosynthesis); Δ2b = C58ΔSpG8‐2b (secondary metabolite biosynthesis); Δ3 = C58ΔSpG8‐3 (siderophore biosynthesis—fabrubactins A and B; iron‐siderophore uptake), Δ4 = C58ΔSpG8‐4 (Ribose transport; monosaccharide catabolism and carbohydrate metabolism); Δ5 = C58ΔSpG8‐5 (opine‐like compounds catabolism); Δ7a = C58ΔSpG8‐7a (environmental signal sensing/transduction); Δ7b = C58ΔSpG8‐7b (environmental signal sensing/transduction). Data were compared by analysis of variances between WT condition and the others one by one. The statistically significant differences are indicated with the symbol * (*** for *p*‐value < 0.001; ** for 0.001 < *p*‐value < 0.01; * for 0.01 < *p*‐value < 0.05).

The PCA comparison of root metabolite profiles of the WT and NI condition showed discrimination between the two along PC1 (Figure 3A). Eleven root metabolites significantly varied in their relative abundances between both conditions. Five of which are underabundant (**8**, **40**, **44**, **87** and **90**), the remaining six are overabundant (**24**, **25**, **26**, **36**, **65** and **79**) in the NI condition compared to the WT condition (Figure 4). Among them, two compounds (**8** and **25**) were exclusively discriminating between the NI and the WT condition (i.e., not discriminating for the mutant strain conditions).

#### 3.4.3 Effect of *A. fabrum* Specific Regions on Root Specialized Metabolite Profiles

Root metabolite profiles of each mutant strain condition were singly compared with the WT condition (Figure 3B–J). All root metabolite profiles from mutant strain conditions clearly separated from that of the WT condition, except for the C58ΔSpG8‐2a mutant strain (Figure 3C). Fifteen chromatographic peaks were found to display a significantly different relative abundance compared to the WT condition for at least one mutant condition, nine of which were highlighted above in the *A. fabrum*— *M. truncatula* root interaction (Figure 4). Remarkably, there were only two patterns of change in metabolite relative abundance: on the one hand, compounds that were exclusively overabundant in mutant strain conditions compared to the WT condition (**24**, **26**, **36**, **65**, **66**, **71** and **79**), and on the other, compounds that were exclusively underabundant in mutant strain conditions (**1**, **40**, **41**, **44**, **50**, **81**, **87** and **90**) (Figure 4). These analyses confirmed a gradient of differences in root metabolite profiles depending on the mutant strain conditions compared to the WT condition (Figures 3 and 4). This gradient ranged from a non‐different profile for plants inoculated with strain C58ΔSpG8‐2a (Figures 3C and 4) to profiles clearly differentiated from the WT condition (for plants inoculated with strains C58ΔSpG8‐3, C58ΔSpG8‐4 and C58ΔSpG8‐7b) by variations in the relative abundance of 8 to 13 metabolites (Figures 3E–J and 4).

### 3.5 Identification or Annotation of Discriminating Metabolites

The UHPLC‐UV/DAD‐MS/MS QTOF data were explored to identify the discriminating compounds highlighted by statistical analyses. Study of the spectral data (UV–vis maxima; accurate mass; MS and MS/MS spectra in positive and negative ionization mode) allowed the annotation and/or the identification of 13 compounds by comparison to bibliographical data and analyses of standard compounds when available. Chemical data of the annotated discriminating compounds are shown in Table 3. The detailed interpretation of these spectral data for the structural elucidation of these compounds was given in the Supporting Information (Figure S7, and additional text 'SupText1'). All of the discriminating metabolites except one are phenolic compounds belonging to the flavonoid family of different subclasses (aurone, flavanone, isoflavone, flavone and flavonol). The other is an amino acid (**1**, tryptophan), whose underabundance was significant in some conditions (Figure 4).

**TABLE 3:** UPHLC‐UV/DAD‐ESI‐MS MS Q‐TOF data of discriminant metabolites in *M. truncatula* root in interaction with *A. fabrum* strains.

No.	RT (min)	UV *λ*_max_ (nm)	UHPLC‐MS Q‐ToF analysis					UHPLC‐MS/MS Q‐ToF analysis		Proposed annotation	ID	References
			IM	Theoretical m/z	Observed ions m/z	Molecular formula	Δppm	CE (V)	Fragments MS/MS (% base peak)			
**1**	2.388	218, 278, 288sh	+	205.097154	205.0969 [M + H]^+^; 227.0789 [M + Na]^+^	C_11_H_12_N_2_O_2_	−1.24	10	146.0593 (100) 188.0697 (74) [M + H‐H_2_O]^+^ 118.0644 (41) indole moiety 205.0851 (5)	Tryptophan	a, c	(Pallerla et al. 2019)
			−	203.082601	203.0824 [M‐H]^−^		−0.99	10	116.0505 (100) indole moiety 203.0826 (68) 142.0669 (27) 159.0918 (17) 186.0553 (4) [M‐H‐H_2_O]^−^			
**8**	4.093	260, 296sh	+		449,1369 [M + H]^+^					Unknown		
**24**	7.332	295, 346sh	+	473.105433	473.1110 [M + Na]^+^	C_21_H_22_O_11_	11.77	20	473.1020 (100) [M + Na]^+^ 311.0519 (28) [M + Na‐hex]^+^	Maesopsin 6‐O‐glucoside	a	(Li et al. 1997)
			−	449.108935	449.1094 [M‐H]−		1.03	30	269.0461 (100) [M‐H‐hex‐H_2_O]^−^ 259.0605 (20) [M‐H‐hex‐CO]^−^ 449.1073 (14) [M‐H]^−^ 287.0579 (5) [M‐H‐hex]^−^			
**25**	7.735	232, 278, 290sh								Unknown		
**26**	8.062	292, 343sh	+	473.105433	473.1035 [M + Na]^+^	C_21_H_22_O_11_	−4.08	30	311.0514 (100) [M + Na‐hex]^+^ 473.1038 (21) [M + Na]^+^ 185 (19)	Maesopsin 4‐O‐glucoside	a, c	(Stochmal et al. 2009)
			−	449.108935	449.1094 [M‐H]^−^		1.03	20	259.0635 (100) [M‐H‐hex‐CO]^−^ 287.0588 (82) [M‐H‐hex]^−^ 269.0483 (78) [M‐H‐hex‐H_2_O]^−^ 449.1132 (65) [M‐H]^−^			
**36**	10.208	248sh, 262, 321sh, 354	+	771.197835	771.1935 [M + H]^+^	C_33_H_38_O_21_	−5.62	20	463.0841 (100) [M + H‐deoxyhex‐hex]^+^ 287.0542 (22) [M + H‐deoxyhex ‐hex‐hexu]^+^ 625.1323 (6) [M + H‐deoxyhex]^+^ 771.1950 (1) [M + H]^+^	Kaempferol O‐hexuronyl‐[desoxyhexose]‐hexoside	a	(Budzianowski 1991)
			−	769.183282	769.1844 [M‐H]^−^		1.45	30	593.1523 (100) [M‐H‐hexu]^−^ 285.0404 (20) [M‐H‐hexu‐hex‐deoxyhex]^−^ 769.1841 (17) [M‐H]^−^			
**39**	10.792	232, 273	+		515,0582 [M + H]^+^					Unknown		
**40**	11.003	228, 252, 324	+	607.129361	607.1271 [M + H]^+^	C_27_H_26_O_16_	−3.72	20	255.0639 (100) [M + H‐hexu‐hexu]^+^ 607.1260 (31) [M + H]^+^ 431.0944 (3) [M + H‐hexu]^+^	7,4′‐dihydroxyflavone dihexuronide	a, b	(Marczak et al. 2016; Singh et al. 2010)
			−	605.114808	605.1149 [M‐H]^−^		0.15	30	351.0571 (100) [M‐H‐Aglycone]^−^ 175.0246 (30) [M‐H‐Aglycone‐hexu]^−^ 253.0514 (15) [M‐H‐2hexu]^−^			
**41**	11.607	216, 228, 276, 310	+	617.147691	617.1441 [M + Na]^+^	C_27_H_30_O_15_	−5.82	40	441.1136 (100) [M + Na‐hexu]^+^ 617.3226 (2) [M + Na]^+^	Liquiritin‐7‐O‐hexuronide	a, b	(Ma et al. 2016)
			−	593.151194	593.1514 [M‐H]^−^		0.35	30	255.0669 (100) [M‐H‐hexu‐hex]^−^ 593.1507 (11) [M‐H]^−^ 417.1188 (2) [M‐H‐hexu]^−^			
**44**	12.523	228, 252, 312sh, 330	+	431.097273	431.0960 [M + H]^+^	C_21_H_18_O_10_	−2.95	20	255.0636 (100) [M + H‐hexu]^+^ 431.0973 (2) [M + H]^+^	7,4′‐dihydroxyflavone‐7‐O‐glucuronide	a, b	(Singh et al. 2010; Staszków et al. 2011)
			−	429.082720	429.0830 [M‐H]^−^		0.65	20	253.0506 (100) [M‐H‐hexu]^−^ 429.0818 (1) [M‐H]^−^			
**50**	14.313	268, 286, 326	+	623.124276	623.1209 [M + H]^+^	C_27_H_26_O_17_	−5.42	20	271.0592 (100) [M + H‐2hexu]^+^ 623.1211 (44) [M + H]^+^ 447.0920 (10) [M + H‐hexu]^+^	Apigenin 4′‐O‐dihexuronide	a, b	(Marczak et al. 2010)
			−	621.109723	621.1103 [M‐H]^−^		0.93	20	351.0574 (100) [M‐H‐Aglycone]^−^ 621.1100 (3) [M‐H]^−^			
**65**	17.992	254, 282sh, 322	+		783.1660 [M + H]^+^	C_37_H_33_O_19_		20	255.0610 (100) [M + H‐hexu‐ferulate‐hexu]^+^ 783.1640 (62) [M + H]^+^ 353.0832 (18) [M + H‐hexu‐Aglycone]^+^ 431.0900 (12) [M + H‐hexu‐ferulate]^+^ 177.0518 (11) [M + H‐2hexu‐Aglycone]^+^	7,4′‐dihydroxyflavone‐O‐[feruloyl‐hexuronyl ‐O‐hexuronide]	a, b	(Kachlicki et al. 2016; Marczak et al. 2010)
			−		781.1711 [M‐H]^−^			20	527.1112 (100) [M‐H‐Aglycone]^−^ 333.0519 (21) [M‐H‐Aglycone‐ferulate]^−^			
**66**	18.328	251, 268, 346	+	477.102753	477.1009 [M + H]^+^	C_22_H_20_O_12_	−3.88	50	286.0456 (100) [M + H‐hexu‐CH_3_]^+^ 301.0707 (57) [M + H‐hexu]^+^ 258.0500 (51)	Diosmetin 3′/7‐O‐hexuronide	a, b	(Ferreres et al. 2014; Li et al. 2016)
			−	475.088200	475.0886 [M‐H]^−^		0.84	30	299.0570 (100) [M‐H‐hexu]^−^ 284.0334 (52) [M‐H‐hexu‐CH_3_]^−^			
**71**	19.517	230, 252, 296	+	431.133659	431.1348 [M + H]^+^	C_22_H_22_O_9_	2.65	50	269.0794 (100) [M + H‐hex]^+^ 431.1348 (2) [M + H]^+^	Formononetin‐7‐O‐glucoside (Ononin)	a, c	(Farag et al. 2007)
			−	475.124585	475.1284 [M‐H + HCOOH]^−^		8.03	50	252.0449 (100) [M‐H‐hex‐HCOO‐CH_3_]^−^ 267.0694 (16) [M‐H‐hex‐HCOOH]^−^ 475.1284 (2) [M‐H + HCOOH]^−^			
**79**	21.305	270, 296sh, 324	+	799.171620	799.1615 [M + H]^+^	C_37_H_34_O_20_	−12.6	20	271.0570 (100) [M + H‐2hexu‐ferulate]^+^ 353.0836 (62) [M + H‐hexu‐Aglycone]^+^ 177.0525 (31) [M + H‐2hexu‐Aglycone]^+^ 447.0876 (25) [M + H‐hexu‐ferulate]^+^ 799.1616 (18) [M + H]^+^	Apigenin‐O‐[feruloyl‐glucuronopyranosyl‐O‐glucuronopyranoside]	a, b	(Marczak et al. 2010)
			−	797.157067	797.1673 [M‐H]^−^		12.84	30	527.1112 (54) [M‐H‐Aglycone]^−^ 269.0490 (42) [Aglycone‐H]^−^ 797.1673 (1) [M‐H]^−^			
**81**	21.642	282, 324	+		563,1482 [M + H]^+^					Unknown		
**87**	24.233	212sh, 224sh, 258, 318	+		547.1517 [M + H]^+^	C_26_H_26_O_13_		50	299.0928 (100) [M + H‐gluc malonate]^+^ 284.0707 (47) [M + H‐gluc malonate‐CH_3_]^+^	Afrormosin‐7‐O‐glucoside‐6′‐O‐malonate	a	(Farag et al. 2007, 2008)
**90**	25.422	220, 274	+		515.6927 [M + H]^+^ 992.4288					Unknown		
			−		990.4068							

*Note:* a, comparison with literature MS, MS/MS and UV data. b, comparison with the aglycone moiety showing identical UV and/or mass data. c, comparison with the pure compound showing identical retention time, UV and mass spectra data.

Abbreviations: CE: Collision energy; deoxyhex, deoxyhexose; hex, hexose; hexu, hexuronic acid; gluc, glucoside; ID, Identification procedure; IM, ionization mode.

### 3.6 Effect of Plant Metabolites on Bacterial Growth

Some of the discriminating metabolites or their aglycones were assessed for their effect on the growth of the WT C58 and the mutant strains. All of these metabolites have an inhibitory effect on the WT strain, with the exception of tryptophan and apigenin. The same growth inhibition profile was observed for the species‐specific mutant strains, except for the C58ΔSpG8‐5 strain, which also exhibited inhibited growth in the presence of apigenin (Figure S8). After growth, among the inhibitory compounds, only a significant degradation of formononetin and ononin was observed in the presence of the WT C58 strain (data not shown).

## 4 Discussion

The aim of this work was to assess the involvement of *A. fabrum*‐specific regions in the interaction between the plant and this bacterial species, and more particularly their influence on plant specialized metabolites. Among them, phenolic compounds are often involved in plant‐bacteria interactions (Wang et al. 2022; Singh et al. 2023) and their content has been shown to be affected in plant response to phytobeneficial and phytopathogenic bacteria (Valette et al. 2019; Nong et al. 2023).

### 4.1 Validation of the Experimental Approach

Functional annotations of the specific gene regions (Table 2) and their expression during the interaction with *M. truncatula* roots (Figure S3) suggested a close connection with the plant (Lassalle et al. 2011). All the mutants for these regions are able to colonize and survive on the roots of *M. truncatula* . A lack of effect of a mutant strain on roots cannot therefore be attributed to the disappearance of the mutant bacterial strain on *M. truncatula* roots. Mutant strains were constructed by replacing the species‐specific regions with a *nptII* cassette enabling the strain to resist kanamycin/neomycin by the synthesis of neomycin phosphotransferase II protein (Lang et al. 2013). The root metabolite content of plants inoculated either by the WT C58 strain (WT1) or by the kanamycin resistant C58 derivative (WT2) showed the weak influence of neomycin phosphotransferase II protein on the plant root metabolites. The *nptII* gene was often used as a selectable marker for the construction of genetically modified organisms with no pleiotropic effects on the transcriptomes of transgenic plants (Miki et al. 2009). Moreover, these results with WT1 and WT2 underline the reproducibility of the plant's metabolism response to inoculation with *A. fabrum* C58 strain. Thus, in the plant experiment, the differences in root metabolite profiles observed with the mutant strains are indeed the result of the absence of a species‐specific region.

### 4.2 Biomass Quantity of *M. truncatula* Unaffected by Root Inoculation With *A. fabrum*

Interactions between plants and microorganisms can have a significant impact on plant physiology, growth and health, either positively or negatively depending on the micro‐organism. Previously, an *A. fabrum* strain (GMI9023, a C58 strain without plasmid) was reported to induce an unexpected phytostimulation on maize with an increase in shoot and root dry biomass after 10 days of co‐culture (Walker et al. 2013). However, after 14 days of co‐culture under our conditions, the biomasses of aerial parts and roots were similar for the inoculated or non‐inoculated *M. truncatula* seedlings. No plant growth promoting rhizobacteria (PGPR)‐type effects were observed in our conditions. Likewise, the amounts of methanolic extract obtained from the roots were similar between all conditions. Nevertheless, the analyses revealed a qualitative modification of these root extracts between the WT condition and the NI or mutant strain conditions.

### 4.3 *A. fabrum* Inoculation Modulates Plant's Flavonoid Profile

**FIGURE 5:**
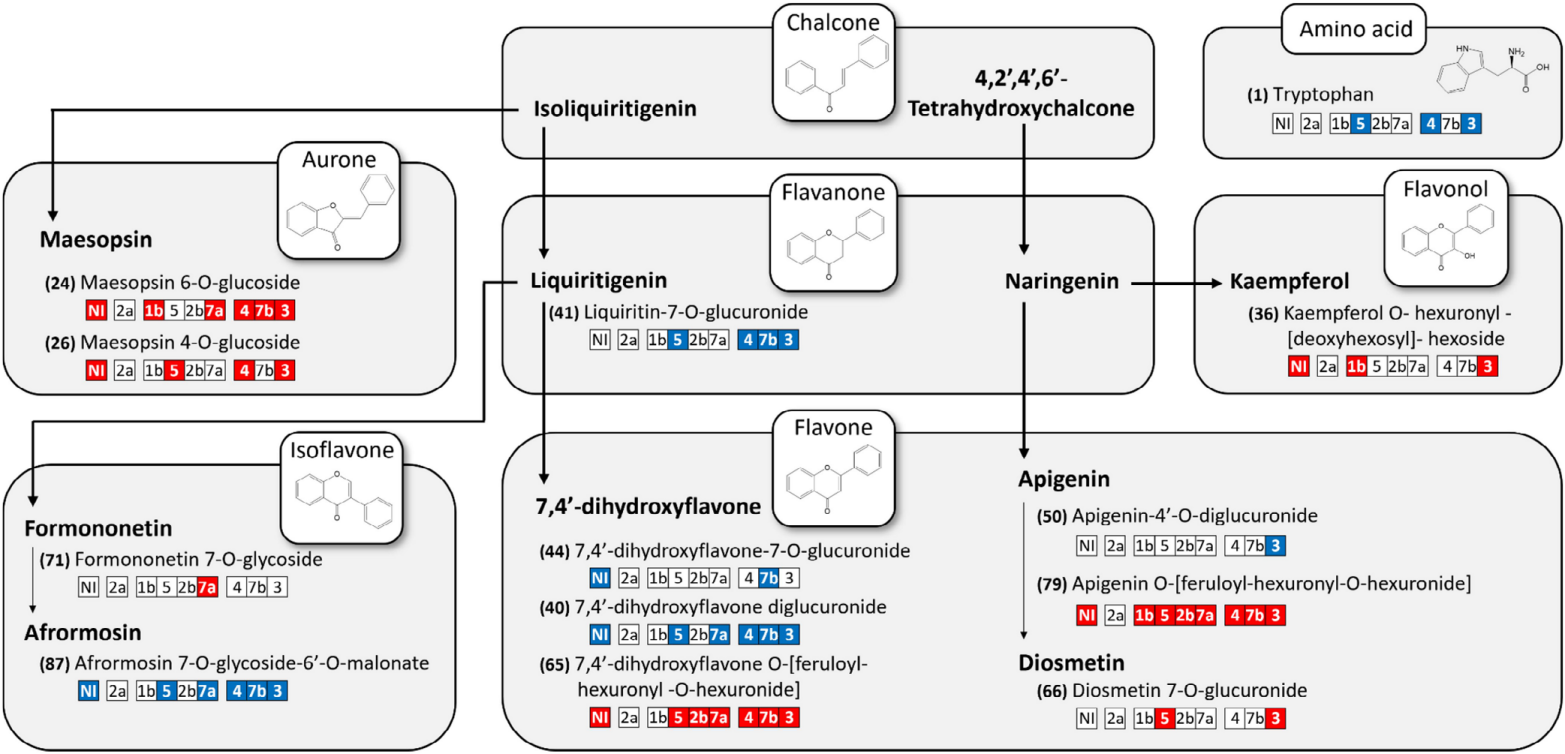
Discriminating metabolites in flavonoid biosynthesis pathways. The numbering in brackets corresponds to metabolite numbering in Table 3 and in heatmap in Figure 4. The discriminating compounds annotated are presented by chemical class and flavonoid subclass and plotted against their biosynthetic pathways in plants. Boxes indicate the experimental conditions of plants inoculated or not with one of the mutant strains of *A. fabrum*. Colours in boxes correspond to the overabundance (red) or underabundance (blue) of each metabolite when compared to the relative abundance of the WT condition. NI: Non‐inoculated condition, numbering 1b to 7b: Plants inoculated with one of the deletion mutants of the *A. fabrum‐*specific regions. 1b = C58ΔSpG8‐1b; 2a = C58ΔSpG8‐2a; 2b = C58ΔSpG8‐2b; 3 = C58ΔSpG8‐3; 4 = C58ΔSpG8‐4; 5 = C58ΔSpG8‐5; 7a = C58ΔSpG8‐7a; 7b = C58ΔSpG8‐7b.

Among the highlighted discriminating metabolites, whatever the conditions, one was an amino acid, while the others were flavonoids belonging to different subclasses (Figure 5). Compared with uninoculated *M. truncatula* plants, the ones challenged by WT C58 strain modulated the relative abundances of 11 compounds among which were identified 8 flavonoids of the flavone (4), isoflavone (1), flavonol (1) and aurone (2) subclasses (Figure 5).

#### 4.3.1 Isoflavone and Flavone Response Upon *A. fabrum* Inoculation

This is in line with other work highlighting the involvement of isoflavone and flavone in plant‐microorganism interactions (Dixon 1986; Dakora and Phillips 1996; Zhang et al. 2007). For example, isoflavones can function as allelopathic agents or as signalling molecules mediating interactions with phytobacteria like *Rhizobium* (Phillips and Kapulnik 1995; Farag et al. 2007). The isoflavone glycoside found as discriminating between the NI and the WT condition is annotated as afrormosin 7‐O‐glucoside‐6″‐O‐malonate (**87**). Afrormosin, afrormosin glucoside and afrormosin glucoside malonate are the major isoflavonoids described in *M. truncatula* cell cultures (Farag et al. 2007, 2008). The malonate conjugated forms can be used to store the less soluble isoflavone aglycones. Upon microbial infection, the aglycones are reported to be generated from the malonate conjugates (Sumner et al. 1996). The inoculation by *A. fabrum* WT C58 strain seems to induce the increase of this molecule (**87**) in roots (78% more in its relative abundance), suggesting an elicitor effect on the biosynthesis and storage of this plant defence compound.

Moreover, some flavones, including 7,4′‐dihydroxyflavone, play a role as signalling molecules in interaction with other organisms. This compound is one of the most potent *nod*‐gene inducers of rhizobia in *M. truncatula* (Wasson et al. 2006; Zhang et al. 2007, 2009). In our study, three of the flavones annotated were hexuronide conjugates of 7,4′‐dihydroxyflavone. The mono‐ and di‐hexuronide derivatives (**40**, **44**) were enhanced in WT condition (by around 56% and 38% more in their relative abundances, respectively) while the feruloyl‐dihexuronide derivative (**65**) was decreased (by around 47% less), as if the feruloyl acylation had less occurred. Similarly, a feruloyl‐dihexuronide derivative of apigenin (**79**) was decreased (by around 61%) in the *M. truncatula* roots inoculated by WT *A. fabrum* C58 strain. Their flavone aglycones (7,4′‐dihydroxyflavone and apigenin) could also be released into the plant's root exudate when it perceives the WT strain, as signalling or defence molecules (Wang et al. 2022). Consequently, less would be stored in the feruloyl‐acylated form. In accordance with this, the 7,4′‐dihydroxyflavone was found to inhibit the growth of the *A. fabrum* WT C58 strain.

#### 4.3.2 Glycosylated Auronol Modulated by *A. fabrum* Inoculation

More surprisingly, two hexoside conjugates of maesopsin (**24**, **26**), which were two isomers of auronols (a small set of aurone derivatives), decrease in the WT condition (by around 57% and 22% in their relative abundances, respectively). Furthermore, both this compound **26** and its aglycone maesopsin have demonstrated an inhibitory effect on the WT bacterial growth (Figure S8). Among plant specialized metabolites, aurones are considered as minor flavonoids due to their low structural diversity (122 described) and limited distribution within plant biodiversity (Bohm 1988; Boucherle et al. 2017). The maesopsin 4‐O‐glucoside (**26**, also named hovetrichoside C) was described in several plant species including *M. truncatula* roots (Boucherle et al. 2017; Stochmal et al. 2009), while maesopsin 6‐O‐glucoside (**24**) seems to have only been isolated from three other plant genera (Bouzidi et al. 2023; Li et al. 1997; Zeng et al. 2015). To our knowledge, these maesopsin hexoside derivatives have not been reported as metabolites involved in plant‐bacteria interactions. However, other compounds belonging to aurones are described as phytoalexins, such as cephalocerone the first discovered (Paré et al. 1992; Boucherle et al. 2017) or hispidol with an antifungal activity against *Phoma medicaginis*, a plant pathogen (Farag et al. 2009). Furthermore, upon yeast elicitation, *M. truncatula* root cell cultures were demonstrated to overproduce two aurones, this hispidol and its glucoside derivative, with a large secretion of hispidol into the culture medium (Farag et al. 2009). These experiments revealed the implication of some peroxidases (MtPRXs) enzymes and isoliquiritigenin as a precursor in their biosynthesis in *M. truncatula* root cell cultures. However, they have never detected them in *M. truncatula* roots and leaves and hypothesize that these hispidols have arisen as by‐products following an imbalance between the upstream and downstream segments of the phenylpropanoid pathway in this cell culture context. In our experiment, not hispidols but two other aurone hexoside derivatives (**24**, **26**) were highlighted. Their decrease in *M. truncatula* roots exposed to WT *A. fabrum* C58 strain could perhaps result from their hydrolysis and the secretion of their aglycone out of the roots in the culture medium (as defensive compounds, like hispidol) or from a limiting availability of isoliquiritigenin more redirected towards the pathways leading to the biosynthesis of certain isoflavones and flavones, like the ones (**87**, **44** and **40**) shown to be increased in parallel in the roots (Figure 5).

### 4.4 Species‐Specific Regions' Involvement in the Plant Metabolite Modifications Upon *A. fabrum* Inoculation

#### 4.4.1 Effect of Most *A. fabrum*‐Specific Regions on Phenolic Compounds in *M. truncatula* Roots

Almost all mutant strains significantly altered the relative abundance of more than one phenolic compound detected in the *M. truncatula* root extracts. In addition, almost all the discriminating root compounds were affected by the inoculation of more than one mutant strain. Among these latter, derivatives of the same aglycones (substituted by malonate, hexose, hexuronic acid, ferulic acid or a combination of these) were significantly modulated by the mutant strains, indicating their impact on certain metabolic pathways during interaction with *M. truncatula* roots, such as the isoflavone, aurone and flavone pathways (Figure 5). Furthermore, the modifications were always in the same way for all the mutant strain conditions concerned (always underabundant or overabundant relative to the WT condition), as if there was a crosstalk or eventually coordinated effect of these specific regions during the interaction of *A. fabrum* with *M. truncatula* (Figures 4 and 5). This is consistent with the previous reports of the coordinated expression by HCA and/or iron of the regions SpG8‐1b and SpG8‐3. Indeed, the presence of the region SpG8‐3 is required for the expression of the SpG8‐1b, and *vice versa* (Baude et al. 2016). To go further in the exploration of these specific gene regions, the putative interactions among the proteins encoded by these specific regions were investigated by applying the STRING v12 database (Figure S9) (Szklarczyk et al. 2023). Some links were observed between proteins from the species‐specific regions. Further analysis could reinforce these findings and provide additional support for the idea that *A. fabrum*‐specific regions may be more closely linked than gene annotations suggest (Lassalle et al. 2011).

#### 4.4.2 The Species‐Specific Region of Curdlan Does Not Impact *M. truncatula* Root Phenolic Compounds

The function of the SpG8‐2a specific genes region of *A. fabrum* is curdlan biosynthesis. This exopolysaccharide (EPS) surrounds bacteria, and its production could likely protect bacteria against environmental stresses and increase its survival in the soil environment (Matthysse 2018). As previously reported by Lasalle et al. the curdlan production by the SpG8‐2a mutant strain was suppressed (Lassalle et al. 2011). However, in our experimental conditions, the SpG8‐2a specific region has no influence on plant metabolites and does not play a direct role in the plant's perception of the bacteria. Interestingly, this region is the only one whose proteins are not directly linked to those of the other specific regions (Figure S9).

#### 4.4.3 Hexuronide Derivatives, a Role in the Adaptation of *A. fabrum* to *M. truncatula* Roots?

Of the thirteen annotated compounds modulated by mutant strains, twelve belong to the flavonoids, eight of which are hexuronide glycoconjugates (Figures 4 and 5). Among the flavonoid glycosides of plants, flavonoid hexuronides are special in that they contain hexuronic acid groups instead of or in addition to sugar units and can also be acylated with hydroxycinnamic acids (HCAs) (Kowalska et al. 2007; Marczak et al. 2010). Glycosylation and acylation have often been reported to modify the bioactivity, stability, solubility, and subcellular localization of phenolic compounds. Glycosylated phenolic compounds are often regarded as storage forms for their aglycones (Le Roy et al. 2016; Essa et al. 2023). A wide diversity of flavonoid hexuronides was reported in roots and aerial parts of *M. truncatula* (Kowalska et al. 2007; Staszków et al. 2011). Furthermore, among the mutant strains involved in the modulation of these flavonoid hexuronides, three (C58ΔSpG8‐3, C58ΔSpG8‐5, or C58ΔSpG8‐7a) showed lower in vitro metabolic activity than the WT strain when grown on medium containing hexuronic acids. The ability to use hexuronic acid could therefore be implicated in the adaptation of *A. fabrum* to *M. truncatula*. However, additional studies are necessary to conclude on their importance in the construction of *A. fabrum* specific niche in the *M. truncatula* rhizosphere rich in hexuronide conjugates.

#### 4.4.4 Feruloyl‐Acylated Flavones Influenced by the Species‐Specific Regions of *A. fabrum*

HCAs and its derivatives are important for *A. fabrum* ecology and fitness in the rhizosphere (Meyer et al. 2018, 2019). The SpG8‐1b specific‐region encodes the complete degradation pathway of some HCAs, which are ultimately used as a source of carbon and energy by *A. fabrum* (Campillo et al. 2014; Meyer et al. 2018). The importance of feruloyl derivatives during the interaction of *A. fabrum* with plants is supported by these results of modulation of feruloyl‐derivatives of flavones. Depending on the mutant strain, the feruloyl‐dihexuronide derivative of 7,4′‐dihydroxyflavone (**65**) and apigenin (**79**) are overabundant compared to the WT condition. Whereas their mono‐ or di‐hexuronide derivatives (**40, 44, 50**) are either less abundant or equivalent under the mutant strain conditions, as if the last step of feruloyl acylation had occurred less in plant roots for the WT condition. The underabundance of flavone feruloyl‐derivatives could be linked to a release of ferulic acid or flavone aglycone into the rhizosphere when the WT strain is perceived by the plant, and/or to a degradation of ferulic acid by the WT strain and not by the mutant strains, whatever the species‐specific region.

#### 4.4.5 Importance of Signals in the Interaction Between *A. fabrum* and Plant

Among the *A. fabrum*‐specific regions, two of them have a greater impact on specialized plant metabolites (Figure 4). The region SpG8‐3 (encoding siderophore biosynthesis) appears to be central among the species‐specific regions, with 13 modified metabolites and its proteins interacting *in silico* with proteins from four other regions (SpG8‐1b, SpG8‐2b, SpG8‐5, and SpG8‐7b). An important function such as iron supply obviously has a wide impact on the physiology of both partners in the interaction.

In contrast, the SpG8‐7b mutant strain modified eight root compounds but its proteins are linked only to proteins from the region SpG8‐3. The SpG8‐7b region is predicted to be involved in the perception of environmental signals. Four of the compounds modified by this C58ΔSpG8‐7b belong to the same flavone biosynthesis pathway involving the 7,4′‐dihydroxyflavone derivatives (**40**, **41**, **44**, **65**), one of them liquiritigenin 4′‐hexosyl‐7‐O‐hexuronide (**41**) being the precursor of the pathway (Figure 5). Liquiritigenin was a nod inducer gene, being then beneficial for the symbiosis legume‐*Rhizobium* (Recourt et al. 1991). Moreover, 7,4′‐dihydroxyflavone is suspected to be a signal molecule (Harrison and Dixon 1993; Wasson et al. 2006; Zhang et al. 2007, 2009). Thus, the SpG8‐7b region could be involved in the signal exchange between the bacteria and the plant. Two proteins from this region (Atu4300 and Atu4305) are homologous to a two‐component system that has been described as being involved in plant host recognition during nodulation or activating specific degradation pathways (Panke et al. 1998; Lang et al. 2008). We suspect a direct role for SpG8‐7b in plant recognition and thus its adaptive advantage in lifestyles that require close interactions with the plant. Given the presumed link between the SpG8‐7b and SpG8‐3 proteins, and the apparent pivotal role of SpG8‐3, it is conceivable that SpG8‐7b is dedicated to the perception of a plant signal that will be transduced and involved in the regulation of SpG8‐3 expression, and through this to other specific regions of *A. fabrum*.

## Author Contributions

**Rosa Padilla:** writing – original draft, formal analysis, investigation, methodology, visualization, software. **Céline Lavire:** conceptualization, writing – review and editing, writing – original draft, project administration, supervision, resources, formal analysis, funding acquisition, validation. **Isabelle Kerzaon:** conceptualization, investigation, funding acquisition, writing – original draft, writing – review and editing, data curation, supervision, formal analysis, software, visualization, validation, methodology, project administration. **Deniz Sarigol:** methodology, visualization, investigation, formal analysis, software. **Thi Huyen Thu Nguyen:** investigation, methodology, visualization, formal analysis. **Florence Dechèvre:** methodology, investigation. **Guillaume Meiffren:** methodology, investigation, validation, data curation. **Ludovic Vial:** conceptualization, investigation, funding acquisition, writing – original draft, writing – review and editing, visualization, validation, formal analysis, supervision, project administration, methodology. **Gilles Comte:** data curation, validation, writing – review and editing. **Vincent Gaillard:** methodology, formal analysis, resources. **Xavier Nesme:** writing – original draft, supervision, project administration, resources, funding acquisition.

## Funding

A part of this work was financially supported by the EcoGenome project of the French Agence Nationale de la Recherche (grant number ANR‐BLAN‐08‐0090) and by the BioEnviS Research Federation. Rosa Padilla received doctoral grants from the French Ministère de l'Education Nationale, de l'Enseignement Supérieur et de la Recherche, and from CONACYT (Consejo Nacional de Ciencia y Tecnología, CVU 397515). This work was also financially supported by the Rhône‐Alpes international cooperation and mobility program (CMIRA) through the award of an internship grant to Thi Huyen Thu Nguyen. This work benefited from the “Serre et Chambres Climatiques” platform, supported by the BioEnviS Research Federation.

## Conflicts of Interest

The authors declare no conflicts of interest.

## Supporting information


**Table S1:** Primers used in this study.
**Figure S1:** Phenotypic changes in deletion mutants compared to wild‐type strains of *A.* *fabrum*.
**Figure S2:** Boxplots illustrating *Medicago truncatula* root colonization by *A. fabrum* wild‐type and deletion mutant strains.
**Figure S3:** *In planta* expression of the *A. fabrum*‐specific regions through the expression of their targeted gene on *M. truncatula* roots.
**Figure S4:** Boxplots illustrating the extraction yield of metabolites from *Medicago truncatula* root inoculated or not by *A. fabrum* wild‐type or deletion mutant strains.
**Figure S5:** A representative UV‐chromatogram (280 nm) obtained by UHPLC‐UV/DAD‐MS QTOF analysis of extracts from *M. truncatula* roots inoculated or not with the wild‐type or the deletion mutant strains of *A. fabrum* C58.
**Figure S6:** PCA score plot of root specialized metabolite profiles of *M. truncatula* seedlings inoculated by the two wild‐type strain conditions.
**Figure S7:** A comparison of the chromatographic and spectral data of a maesopsin 4‐O‐glucoside standard with that of compounds **24** and **26** in the *Medicago truncatula* (*Mt*) root extract.
**Figure S8:** Effect of some plant metabolites on *A. fabrum* bacterial growth.
**Figure S9:** Protein–protein interaction network of the *A. fabrum* C58 species‐specific proteins.
**SupText1:** Detailed interpretations for identifying or annotating discriminating metabolites.

## Data Availability

The data that supports the findings of this study are available in the Supporting Information of this article.
