# Supplementary material for: Most of the Specific Genomic Regions of Agrobacterium fabrum Shape Phenolics Content of Medicago truncatula Roots
Source: Environ Microbiol Rep. 2026 Aug 7;18(4):e70394. doi: 10.1111/1758-2229.70394 (PMC13451346; doi:10.1111/1758-2229.70394)
Supplement: Supplementary file 1 [file EMI4-18-e70394-s001.docx]

**Supporting Information**

**Most of the specific genomic regions of *Agrobacterium fabrum* shape phenolics content of *Medicago truncatula* roots**

Rosa PADILLA^1,^*****, Ludovic VIAL^1,^*****, Florence DECHÈVRE^1^, Thi Huyen Thu NGUYEN^1,#^ , Deniz SARIGOL^1^, Vincent GAILLARD^1^, Guillaume MEIFFREN^1^, Gilles COMTE^1^, Xavier NESME^1^, Céline LAVIRE^1^*****, Isabelle KERZAON^1^***** **^🖂^**.

^1^ Université Lyon 1, CNRS, INRAE, LEM, UMR 5557, UMR 1418 Villeurbanne, France

***** These authors equally contributed to this work.

**🖂** Corresponding author at: UMR5557 Université Lyon 1/CNRS - Écologie Microbienne, 6 rue R. Dubois, Bâtiment Forel 5ème étage, Centre d’Etude des Substances Naturelles (CESN), 69622 Villeurbanne Cedex, France.

*E-mail address:* isabelle.kerzaon@univ-lyon1.fr (Isabelle Kerzaon)

*^#^ Present address: Department of Life Sciences, University of Science and Technology of Hanoi, Vietnam Academy of Science and Technology,18 Hoang Quoc Viet, Cau Giay, Hanoi 10072, Vietnam; Saint Paul Hospital, 12 Chu Van An, Hanoi 11114, Vietnam*

**Keywords:** *Agrobacterium*, metabolomic, plant-bacteria interaction, specific genes, phenolic compounds, Medicago, root metabolites

**TABLE S1. Primers used in this study**

| *A. fabrum*-specific region | Analysis and region | Forward primer (5’ to 3’) | Reverse primer (5’ to 3’) | Reference |
| --- | --- | --- | --- | --- |
|  |  |  |  |  |
| **Transcriptional fusions** | | | |  |
| SpG8-1b | *Atu1416* promoter |  |  | (Meyer et al., 2018) |
| SpG8-2a | *atu3057* promoter | AAGCTTTGACGGTTGTGAACAGCACT | GAGGTGAATACAGGCGGAAA | This study |
| SpG8-2b | *atu3073* promoter | AAGCTTACTGAAACCGACATGAACGC | CCGCCGAGAATTCCGATAGT | This study |
| SpG8-3 | *atu3675* promoter | AAGCTTAAATCGCGTTCTCCAGATGG | GGGTCTCTACTGGCTCGATG | This study |
| SpG8-4 | *atu3817* promoter | TGGTTCCCAGACGTCGTTTA | CAGTTTGATGTAGCGAGCCA | This study |
| SpG8-5 | *Atu3948* promoter | AAGCTTGAAGGTCGGTGGCATTGT | ACGGCTCTTGCTTGTTCG | This study |
| SpG8-7a | *atu4292* promoter | ATCGATGTGCAGAGCTTGCTGACG | CAGGGTGATGTGGAGATCGT | This study |
| SpG8-7b | *atu4299* promoter | AAGCTTCGAGCCATTTCATGAGTGCT | AGGAGACAATGCAACCCGTA | This study |
| Insertion verification in pGEMT | | GTTTTCCCAGTCACGAC | CAGGAAACAGCTATGAC | Promega |
| Insertion verification in pOT1e | | CGGTTTACAAGCATAAAGC | CATTTTTTCTTCCTCCACTAG | (Pothier et al., 2007) |
|  |  |  |  |  |
| **Construction of the *A. fabrum*-specific regions deletion mutants (inactivation gene clusters)** | | | |  |
| ΔSpG8-1b | Upstream region of *atu1409* | GATGAAGCCGTCAACATCC | AGATCGGTGACGGAGAAG | (Lassalle et al., 2011) |
|  | Downstream region of *atu1423* | TCCCTCCGGATGAGAACG | GGAGGTGGTCCATGATGT |  |
| ΔSpG8-2a | Upstream region of *atu3054* | CTGAATTTTGCGGATGAGC | TATTCGTCGGTTCCTGCG | (Lassalle et al., 2011) |
|  | Downstream region of *atu3059* | TCCCTCGGAAGTCCGCTT | GTTGCCTCGCTGATGATG |  |
| ΔSpG8-2b | Upstream region of *atu3069* | CCGAGTGTGATGATGACGAG | CATGGTGAAGGCAGTTGCTA | This study |
|  | Downstream region of *atu3073* | TTCCATAAAGCCCTCATTGG | GTTCGACGAGTTGATGACGA |  |
| ΔSpG8-3 | Upstream region of *atu3663* | CCGGTTCTACATCCTGGAAA | GACAACATGCCCCTCTCCTA | (Baude et al., 2016) |
|  | Downstream region of *atu3693* | CCTGCTCAACAGGCTACTCC | TCTGGAACGTCACCGACATA |  |
| ΔSpG8-4 | Upstream region of *atu3808* | ATGACCAGTTGCCAGACCTC | TGATCCGCAGGCCTATATTC | This study |
|  | Downstream region of *atu3830* | GTTGACGGCAATATCGTGTG | AGCCAGCAGGATTTTGAGAA |  |
| ΔSpG8-5 | Upstream region of *atu3947* | CGTTATCGCTGATCCCATTT | GCTGCCTATTTCCTGTCCAA | This study |
|  | Downstream region of *atu3952* | CCTTAACGCAGCTCTTGTCC | CGTGAACCATTTTTGCATTG |  |
| ΔSpG8-7a | Upstream region of *atu4285* | GATCGACGAATTGTCGCG | GGCCTGTTGATCTACAGG | This study |
|  | Downstream region of *atu4294* | GGGTATCGATAGACGAAG | TCGAATGTCCGAGGATGG |  |
| ΔSpG8-7b | Upstream region of *atu4295* | TGCAGCTTGCGATCAATTAC | GCTGAACGAGGTGAAGGAAG | This study |
|  | Downstream region of *atu4307* | CAAGCTCCTGACCGACTTTC | GCTGAACGAGGTGAAGGAAG |  |

**FIGURE S1.**

**Phenotypic changes in deletion mutants compared to wild-type strains of *A. fabrum*.**

Metabolic activity of specific-region deletion mutant strains C58ΔSpG8-3 (= Δ3; **△**), C58ΔSpG8-5 (= Δ5; **+**) and C58ΔSpG8-7a (= Δ7a; **◊**) was compared to wild-type strains WT1 (⬤) and WT2 (**◯**; see **Table 2** for more information on strains) by using Biolog phenotype microplates. Metabolic activities of the mutant strains are the same as the wild-type strains when grown on succinate (**A**) but differ when grown on hexuronic acids: D-galacturonic acid (**B**), gluconic acid (**C**) and D-glucuronic acid (**D**). No difference observed for other carbon sources tested on these microplates (Biolog phenotype MicroArrays PM1 and PM2A).

**FIGURE S2.**

**Boxplots illustrating *Medicago truncatula* root colonization by *A. fabrum* wild-type and deletion mutant strains.**

For each bacterial strain, the emerging root from seeds of *M. truncatula* *in vitro* cultivated was inoculated by 5 µL of a suspension at 2.10^5^ bacteria/mL. Bacteria abundance on roots was determined 14 dpi and expressed as log10 CFU (colony-forming units) per gram of root dry weight. Boxes cover 50% of the data. Central lines represent the medians and whiskers represent the minimum and maximum values among non-atypical data. The cross (𐌢) denotes the mean value of the data with at least three replicates. Some similar levels of root colonization (mean values from 9.0 to 10.3 log10 CFU per gram of root dry weight) were observed between the wild-type (C58) strain and all the mutant strains (Δ1b = C58ΔSpG8-1b; Δ2a = C58ΔSpG8-2a ; Δ2b = C58ΔSpG8-2b ; Δ3 = C58ΔSpG8-3, Δ4 = C58ΔSpG8-4 ; Δ5= C58ΔSpG8-5 ; Δ7a = C58ΔSpG8-7a ; Δ7b= C58ΔSpG8-7b). No statistically significant differences were observed (Kruskal-Wallis test, *p* = 0.5026).

**FIGURE S3.**

***In planta* expression of the *A. fabrum*-specific regions through the expression of their targeted gene on *M. truncatula* roots.**

**A.** Expression of SpG8-1b region, represented by expression of *atu1416* gene (*e.g.* transcriptional fusion between *atu1416* promoter region and eGFP gene). **B.** Expression of SpG8-2a region, represented by expression of *atu3057* gene. **C.** Expression of SpG8-2b region, represented by expression of *atu3073* gene. **D.** Expression of SpG8-3 region, represented by expression of *atu3675* gene. **E.** Expression of SpG8-4 region, represented by expression of *atu3817* gene. **F.** Expression of SpG8-5 region, represented by expression of *atu3948* gene. **G.** Expression of SpG8-7a region, represented by expression of *atu4292* gene. **H.** Expression of SpG8-7b region, represented by expression of *atu4299* gene. Gene expression was monitored by confocal microscopy using transcriptional fusions at 14 dpi. Representative pictures from five plants per specific region are shown. Green fluorescence showed bacteria able to express the corresponding transcriptional fusion. The green autofluorescence of the plants helped to distinguish plant cells. All transcriptional fusions are induced in contact with the entire *M. truncatula* root system.

**FIGURE S4.**

**Boxplots illustrating the extraction yield of metabolites from *Medicago truncatula* root inoculated or not by *A. fabrum* wild-type or deletion mutant strains.**

For each condition 14 dpi, the roots of *M. truncatula* *in vitro* cultivated were harvested, dried and extracted with methanol. Root crude extracts were weighed and expressed as mg of crude extract per mg of root dry mass. Boxes cover 50% of the data. Central lines represent the medians and whiskers represent the minimum and maximum values among non-atypical data. The cross (𐌢) denotes the mean value of the data (n=4 or 5). Similar amounts of crude extract were obtained from roots (mean values ± SE, from 0.21 ± 0.01 to 0.33 ± 0.08 mg of crude extract per mg of root dry mass), whether *M. truncatula* seedlings were non-inoculated (NI) or inoculated with the wild-type C58 (WT1, WT2) or the deletion mutant strains (Δ1b = C58ΔSpG8-1b; Δ2a = C58ΔSpG8-2a; Δ2b = C58ΔSpG8-2b; Δ3 = C58ΔSpG8-3; Δ4 = C58ΔSpG8-4; Δ5= C58ΔSpG8-5; Δ7a = C58ΔSpG8-7a; Δ7b= C58ΔSpG8-7b). No statistically significant differences were observed (Kruskal-Wallis test, *p* = 0.1023).

**FGURE S5.**

**A representative UV-chromatogram (280 nm) obtained by UHPLC-UV/DAD-MS QTOF analysis of extracts from *M. truncatula* roots inoculated or not with the wild-type or the deletion mutant strains of *A. fabrum* C58.**

The integrated chromatographic peaks were considered in the metabolite profiling data study (92 peaks). Peak numbering corresponds to metabolite numbering in **Table 3** and in the heatmap in **Figure 4**. Peaks with an arrow are discriminating compounds with a significant difference (p<0.05, Student t-test) between plants inoculated with the wild-type strain (WT condition) compared to the other conditions (the non-inoculated condition and/or the deletion mutants of the *A. fabrum*-specific regions conditions). Peaks in blue correspond to the underabundant compounds, peaks in red correspond to the overabundant compounds compared to the WT condition.

**FIGURE S6.**

**PCA score plot of root specialized metabolite profiles of *M. truncatula* seedlings inoculated by the two wild-type strain conditions.**

PCA were performed on the data matrix from chromatographic data at 280 nm obtained from each methanolic extract of *M. truncatula* roots based on peak areas and retention time. Plants were inoculated with *A. fabrum* C58 wild-type strain. WT1: plants inoculated with the wild-type strain, WT2: plants inoculated with the wild-type strain carrying the *ntpII* kanamycin resistance gene. This comparison by PCA did not show any separation of *M. truncatula* root metabolite profiles between these two WT conditions.

**FIGURE S7. A comparison of the chromatographic and spectral data of a maesopsin 4-O-glucoside standard with that of compounds 24 and 26 in the *Medicago truncatula* (*Mt*) root extract. A.** Extracted ion chromatogram in negative ionization mode at *m/z* 449.1100 which corresponds to the [M-H]- adduct of maesopsin 4-O-glucoside. It shows that the retention time of compound **26** is similar to that of the standard. **B.** UV-vis spectra, **C.** MS² spectra in positive ionization mode, **D.** MS² spectra in negative ionization mode of maesopsin 4-O-glucoside, compound **24** and **26** detected in *Mt* root extract.

**FIGURE S8. Effect of some plant metabolites on *A. fabrum* bacterial growth** *(legend on the next page)*

**FIGURE S8. Effect of some plant metabolites on *A. fabrum* bacterial growth**

Bacteria were inoculated in AB medium at OD_600nm_ = 0.025 with or without the addition of one compound: tryptophan, liquiritigenin, kaempferol, ononin, diosmetin, maesopsin, maesopsin 4-O-glucoside (for C58 WT only), formononetin or 7,4’ -dihydroxyflavone at 500 µM or apigenin at 200 µM. Bacterial growth (OD_600nm_) was monitored in triplicate in Bioscreen plates using Bioscreen plate reader (28°C, shaking every 20 min before measurement). The experiment was done in two biological replicates. Bacterial growth data were analyzed with a linear mixed-effects modeling (LMM) approach to account for the longitudinal nature of the data. To capture non-linear growth kinetics, time was modeled using natural cubic splines (df = 4). The statistical differences between the Control (with dH_2_O) and molecules conditions are represented (**: *p* < 0.01; ***: *p* < 0.001). The bacteria were: WT = wild-type C58; Δ1b = C58ΔSpG8-1b; Δ2a = C58ΔSpG8-2a; Δ2b = C58ΔSpG8-2b; Δ3 = C58ΔSpG8-3; Δ4 = C58ΔSpG8-4; Δ5= C58ΔSpG8-5; Δ7a = C58ΔSpG8-7a; Δ7b= C58ΔSpG8-7b.

**FIGURE S9.**

**Protein–protein interaction network of the *A. fabrum* C58 species-specific proteins.**

The interactions among the proteins encoded by the *A. fabrum* C58 species-specific regions were explored by applying the STRING v12 database (https://string-db.org/), which systematically collects and integrates protein-protein interactions, either physical ones or functional associations (Szklarczyk et al., 2023). The network was constructed using a high confidence level of 0.7. The proteins are represented by nodes, whose color depends on the species-specific region they belong to: red for SpG8-1b, dark grey for SpG8-2a, yellow for SpG8-2b, cyan for SpG8-3, orange for SpG8-4, green for SpG8-5, magenta for SpG8-7a and purple for SpG8-7b. The interactions among them are represented by edges. In white, there are three proteins that do not belong to the species-specific regions but enable the connection between two of them. Proteins from species-specific regions that are not connected are not represented.

**SupText1.**

**Detailed interpretations for identifying or annotating discriminating metabolites.**

The detailed interpretations of the spectral data for the structural elucidation or annotation of the discriminating compounds are given below.

**Amino acid.** The polar compound **1** that eluted at 2.4 min had an [M+H]^+^ ion at *m/z* 205.0951 which is consistent with the molecular formula C_11_H_12_N_2_O_2_. In MS² mode, it produced ions at *m/z* 188.0697 [M+H-H_2_O]^+^ indicating the loss of a water molecule and at *m/z* 118.0644 [M+H-87]^+^ which corresponds to an indole moiety according to its accurate mass. A comparison of spectral data with literature data (Jiang et al., 2011; Pallerla et al., 2019) and authentic standard analysis led to the identification of this compound as **tryptophan**.

**Flavonoids. Aurones.** Compounds **24** and **26**, eluted respectively at 7.33 and 8.06 min, had identical UV-vis spectra and similar accurate mass at *m/z* 449.1094 corresponding to [M-H]^-^ ions that both fragmented to give product ions at *m/z* 287 [M-H-162]^-^, 269 [M-H-162-18]^-^, 259 [M-H-162-28]^-^, indicating the loss of an hexose followed by the loss of -H_2_O or –CO respectively. Comparison to literature data (Stochmal et al., 2009; Ye et al., 2009; Yoshikawa et al., 1998) led us to propose these compounds as maesopsin glycosides. The analysis of an authentic standard allowed the unequivocal identification of **26** as **maesopsin 4-O-glucoside** (**Figure S7**). Compound **24** was tentatively identified as an isomer as **maesopsin 6-O-glucoside** (Bouzidi et al., 2023; Li et al., 1997).

**Flavonoids. Flavonol.** Compound **36** was observed as an ion [M+H]^+^ at *m/z* 771.1950 that yielded fragments at *m/z* 625.1323 [M+H-146]^+^, 463.0841 [M+H-146-162]^+^ and 287.0542 [M+H-146-162-176]^+^ indicating the losses of a deoxyhexose, of a [deoxyhexose + hexose] and of a [deoxyhexose + hexose + hexuronic acid], respectively. The negative MS² spectra showed an ion [M-H]^-^ at *m/z* 769,1841 giving a daughter ion at *m/z* 593,1523 [M-H-176]^-^ indicating the loss of a hexuronic acid and another product ion at *m/z* 285,0404 [M-H-146-162]^-^ indicating a concomitant loss of a hexose and a deoxyhexose. The UV-vis spectrum indicated a kaempferol as aglycone. These data and the literature led to annotate this compound as a **kaempferol O-hexuronyl-[deoxyhexosyl]-hexoside** (Budzianowski, 1991; Nosrati Gazafroudi et al., 2024), but exact position and nature of the three substituents could not be determined.

**Flavonoids. Flavanone.** Compound **41** was observed as an ion [M-H]^-^ at *m/z* 593.1514 yielding in MS² a minor ion at *m/z* 417.1188 [M-H-176]^-^ indicating the loss of a hexuronic acid and a major product ion at *m/z* 255.0669 [M-H-176 -162]^-^ indicating the loss of a hexuronic acid and a hexose with an accurate mass consistent with the aglycone liquiritigenin. Therefore, combined to literature data (Ma et al., 2016; Qiao et al., 2012; Zhou et al., 2017) and the aglycone standard analyses, this compound was tentatively identified as **liquiritin-7-O-hexuronide**.

**Flavonoids. Flavone.** Compound **40** was tentatively annotated as a **7,4’-dihydroxyflavone dihexuronide** according to the UV-vis spectrum, fragmentation pattern, literature data (Marczak et al., 2016; Park et al., 2003; Singh et al., 2010) and aglycone standard analyses. Indeed, the fragmentation of the [M+H]^+^ ion at *m/z* 607.1271 yielded product ions at *m/z* 431.0944 [M+H-176]^+^ and at *m/*z 255.0639 [M+H-176-176]^+^ indicating successive losses of two hexuronic acids and leading to the fragment aglycone corresponding to 7,4’-dihydroxyflavone (consistent with the UV-vis spectrum). The MS² negative spectrum showed a product ion at *m/z* 351.0571 corresponding to the dihexuronide moiety after the loss of the aglycone, leading to the conclusion that the two hexuronic acids were branched together, in accordance with literature data on similar compounds (Marczak et al., 2016). Finally, the hexuronidation on 4’-hydroxyl group of the aglycone was suggested by the hypsochromic shift observed in band I of the UV spectrum (Singh et al., 2010). To our knowledge, this flavone derivative is reported for the first time in the plant kingdom. NMR analysis is required to definitively characterize both sugar moiety and the entire compound.

Compound **44** was observed as an ion [M-H]^-^ at *m/z* 429.0830 giving in MS² a product ion at *m/z* 253.0506 [M-H-176]^-^ by losing a hexuronic acid. Spectral data consistent with literature and the aglycone standard analysis led to annotate this compound as **7-4’-dihydroxyflavone 7-O-glucuronide** (Saleh et al., 1982; Singh et al., 2010; Staszków et al., 2011) already described in some *Medicago* species.

Compound **65** was annotated as **7,4’-dihydroxyflavone-O-[feruloyl-hexuronyl-O-hexuronide]** according to the UV-vis spectrum, fragmentation pattern and aglycone standard analysis. Indeed, the [M+H]^+^ ion at *m/z* 783.1660 yielded two fragments ions at *m/z* 431 [M+H-352]^+^ and at *m/*z 353.0832 [M+H-430]^+^ corresponding to the fragmentation of the molecule in two parts: the aglycone 7,4’-dihydroxyflavone with a hexuronic acid substituent for the first, and a ferulate susbstituted with a hexuronide moiety for the second. A loss of one hexuronic acid was observed from each of these ions, giving product ions at *m/z* 255.0610 [M+H-352-176]^+^ (the aglycone) and 177.0518 [M+H-430-176]^+^ (the ferulate). The MS² negative spectrum showed a product ion at *m/z* 527.1112 [M-H-254]^-^ corresponding to the loss of the aglycone leading to the conclusion that the two hexuronides and ferulate moiety were all linked together (Kachlicki et al., 2016; Marczak et al., 2010). The other product ion at *m/z* 333.0519 [M-H-254-194]^-^ (loss of aglycone and ferulate) demonstrated that the two hexuronides were connected to each other. To our knowledge, this type of flavone derivative was reported for the first time in the plant kingdom. NMR analysis is required to definitively characterize both sugar moiety and the entire compound.

Compound **50** was observed as an ion [M+H]^+^ at *m/z* 623.1209, consistent with the molecular formula C_27_H_26_O_17._ MS² positive spectrum showed daughter ions at *m/z* 447.0920 [M-H-176]^+^ and 271.0592 [M-H-176-176]^+^ indicating successive losses of hexuronide moieties. These data and the UV-vis spectrum indicated an apigenin as aglycon. Furthermore, in MS² negative spectrum, the formation of a product ion at *m/z* 351.0574 resulting from the loss of 270amu (the aglycone apigenin) showed that the two hexuronides are attached to each other (Marczak et al., 2010). A hypsochromic shift was observed in band I of the UV-vis spectrum compared to that of the apigenin, which seemed to show that the substituent is on the nucleus B. Thus, the glucuronidation must be on the 4’-hydroxyl group of apigenin (Mabry et al., 1970). Indeed, the apigenin 7-O-diglucuronide already described in *Medicago truncatula* (Jasiński et al., 2009) was found in our extracts at another retention time of 13.24 min (corresponding to compound **47**). Its UV-vis spectrum, however, showed an λ_max_ at 266 and at 340 nm, indicating a substitution occurring in ring A of the aglycone, unlike compound **50** (Stochmal et al., 2001). Therefore, this data combined to literature data and the aglycone standard analysis, led to tentatively identify this compound as **apigenin 4’-O-dihexuronide**. To our knowledge, this compound is described for the first time in the plant kingdom. NMR analysis is needed to confirm the structure and precise the configuration of this compound.

Compound **66** was observed as an ion [M-H]^-^ at *m/z* 475.0905 consistent with the molecular formula C_22_H_20_O_12_. MS² spectrum showed fragment ions at *m/z* 299.0570 [M-H-176]^-^ and at *m/z* 284.0334 [M-H-176-15]^-^ indicating the loss of an hexuronic acid followed by a loss of -CH_3_ from the aglycone, a diosmetin according to UV-vis spectrum. Data obtained compared to literature data (Li et al., 2016; Ferreres et al., 2014) and the aglycone standard analysis led us to annotate this compound as a **diosmetin-O-hexuronide**. However, the exact position of the substituent (on 3’- or 7-hydroxyl group) could not be determined.

Compound **79** showed a UV-vis spectrum leading to suspecting an apigenin aglycone acylated with an hydroxycinnamic acid. In MS spectra, it was observed as a protonated ion at *m/z* 799.1615 [M+H]^+^, which was further fragmented in MS² producing a series of daughter ions at *m/z* 447.0876 [M+H-352]^+^ (loss of hexuronic acid and ferulate), *m/z* 353.0836 [M+H-446]^+^ (loss of hexuronic acid and apigenin), *m/z* 271.0570 [M+H-352-176]^+^ (ion of apigenin aglycone) and *m/z* 177.0525 [M+H-446-176]^+^ (ion of ferulate moiety). The same fragmentation pattern was described in the literature for a compound detected in leaf extract of *Medicago truncatula* (Fu and Wang, 2015; Jasiński et al., 2009; Marczak et al., 2010)*.* This compound was annotated as an **apigenin O-[feruloyl-glucuronopyranosyl-O-glucuronopyranoside]**.

**Flavonoids. Isoflavone.**

Compound **71** was observed as an ion [M+H]^+^ at *m/z* 431.1348 consistent with the molecular formula C_22_H_22_O_9_. It yielded in positive MS² a product ion at *m/z* 269.0794 [M+H-162]^+^ which revealed the loss of an hexose from an aglycone which could be a formononetin according to the λ_max_ of the UV-vis spectrum (230, 252 and 296 nm) (Farag et al., 2007; Jung et al., 2013; Wen et al., 2007). The negative MS^2^ spectrum showed a deprotonated ion at *m/z* 475.1284 [M-H+HCOO]^-^ that fragmented to give ions at *m/z* 267.0694 [M-H-162]^-^ corresponding to a loss of a hexose and the formic acid and at *m/z* 252.0449 [M-H-162-15]^-^ indicating the loss of the -CH_3_ from the aglycone formononetin. Analysis of the standard compound ononin confirmed the identification of this **formononetin-7-O-glucoside**, besides being already described in *M. truncatula* cell suspension cultures (Farag et al., 2007).

Compound **87** showed in the positive MS spectrum an ion[M+H]^+^ at *m/z* 547.1517 consistent with the molecular formula C_26_H_26_O_13_. MS² spectrum revealed product ions at *m/z* 299 [M-H-162-86]^+^ and 284 [M-H-162-86-15]^+^corresponding to a loss of a glucoside malonate and the loss of the -CH_3_ from the aglycone afrormosin, consistent with the molecular formula and UV-vis spectrum. All these data compared to literature led us to annotate this compound as **afrormosin-7-O-glucoside-6’-O-malonate**, already described in *M. truncatula*  (Farag et al., 2007, 2008; Zhang et al., 2007, 2014).

**References cited in the Supporting Information (SupText1)**

**Baude, J., Vial, L., Villard, C., Campillo, T., Lavire, C., Nesme, X., and Hommais, F.** (2016). Coordinated regulation of species-specific hydroxycinnamic acid degradation and siderophore biosynthesis pathways in *Agrobacterium fabrum*. Appl. Environ. Microbiol. **82**: 3515–3524.

**Bouzidi, A., Azizi, A., Messaoudi, O., Abderrezzak, K., Vidari, G., Hellal, A.N., and Patel, C.N.** (2023). Phytochemical analysis, biological activities of methanolic extracts and an isolated flavonoid from Tunisian *Limoniastrum monopetalum* (L.) Boiss: an *in vitro* and *in silico* investigations. Sci. Rep. **13**: 19144.

**Budzianowski, J.** (1991). Six flavonol glucuronides from *Tulipa gesneriana*. Phytochemistry **30**: 1679–1682.

**Farag, M.A., Huhman, D.V., Dixon, R.A., and Sumner, L.W.** (2008). Metabolomics reveals novel pathways and differential mechanistic and elicitor-specific responses in phenylpropanoid and isoflavonoid biosynthesis in *Medicago truncatula* cell cultures. Plant Physiol. **146**: 387–402.

**Farag, M.A., Huhman, D.V., Lei, Z., and Sumner, L.W.** (2007). Metabolic profiling and systematic identification of flavonoids and isoflavonoids in roots and cell suspension cultures of *Medicago truncatula* using HPLC-UV-ESI-MS and GC-MS. Phytochemistry **68**: 342–354.

**Ferreres, F., Grosso, C., Gil-Izquierdo, A., Valentão, P., Azevedo, C., and Andrade, P.B.** (2014). HPLC-DAD-ESI/MSn analysis of phenolic compounds for quality control of *Grindelia robusta* Nutt. and bioactivities. J. Pharm. Biomed. Anal. **94**: 163–172.

**Fu, F. and Wang, H.L.** (2015). Metabolomics reveals consistency of the shoot system in *Medicago truncatula* by HPLC-UV-ESI-MS/MS. Int. J. Food Sci. Technol. **50**: 2183–2192.

**Jasiński, M., Kachlicki, P., Rodziewicz, P., Figlerowicz, M., and Stobiecki, M.** (2009). Changes in the profile of flavonoid accumulation in *Medicago truncatula* leaves during infection with fungal pathogen *Phoma medicaginis*. Plant Physiol. Biochem. **47**: 847–853.

**Jiang, P., Dai, W., Yan, S., Chen, Z., Xu, R., Ding, J., Xiang, L., Wang, S., Liu, R., and Zhang, W.** (2011). Potential biomarkers in the urine of myocardial infarction rats: a metabolomic method and its application. Mol. Biosyst. **7**: 824–831.

**Jung, J.-Y., Jung, Y., Kim, J.-S., Ryu, D.H., and Hwang, G.-S.** (2013). Assessment of peeling of *Astragalus* roots using 1H NMR- and UPLC-MS-based metabolite profiling. J. Agric. Food Chem. **61**: 10398–10407.

**Kachlicki, P., Piasecka, A., Stobiecki, M., and Marczak, Ł.** (2016). Structural characterization of flavonoid glycoconjugates and their derivatives with mass spectrometric techniques. Mol. Basel Switz. **21**: 1494.

**Lassalle, F. et al.** (2011). Genomic species are ecological species as revealed by comparative genomics in *Agrobacterium tumefaciens*. Genome Biol. Evol. **3**: 762–781.

**Li, Q., Liu, Y., Han, L., Liu, J., Liu, W., Feng, F., Zhang, J., and Xie, N.** (2016). Chemical constituents and quality control of two *Dracocephalum* species based on high-performance liquid chromatographic fingerprints coupled with tandem mass spectrometry and chemometrics. J. Sep. Sci. **39**: 4071–4085.

**Li, X.-C., Cai, L., and D.Wu, C.** (1997). Antimicrobial compounds from *Ceanothus americanus* against oral pathogens. Phytochemistry **46**: 97–102.

**Ma, H., Liu, Y., Mai, X., Liao, Y., Zhang, K., Liu, B., Xie, X., and Du, Q.** (2016). Identification of the constituents and metabolites in rat plasma after oral administration of HuanglianShangqing pills by ultra high-performance liquid chromatography/quadrupole time-of-flight mass spectrometry. J. Pharm. Biomed. Anal. **125**: 194–204.

**Mabry, T., Markham, K.R., and Thomas, M.B.** (1970). The Systematic Identification of Flavonoids (Springer-Verlag: Berlin Heidelberg).

**Marczak, Ł., Stobiecki, M., Jasiński, M., Oleszek, W., and Kachlicki, P.** (2010). Fragmentation pathways of acylated flavonoid diglucuronides from leaves of *Medicago truncatula*. Phytochem. Anal. **21**: 224–233.

**Marczak, Ł., Znajdek-Awiżeń, P., and Bylka, W.** (2016). The use of mass spectrometric techniques to differentiate isobaric and isomeric flavonoid conjugates from *Axyris amaranthoides*. Mol. Basel Switz. **21**: 1229.

**Meyer, T., Renoud, S., Vigouroux, A., Miomandre, A., Gaillard, V., Kerzaon, I., Prigent-Combaret, C., Comte, G., Moréra, S., Vial, L., and Lavire, C.** (2018). Regulation of hydroxycinnamic acid degradation drives *Agrobacterium fabrum* lifestyles. Mol. Plant. Microbe Interact. **31**: 814–822.

**Nosrati Gazafroudi, K., Mailänder, L.K., Daniels, R., Kammerer, D.R., and Stintzing, F.C.** (2024). From stem to spectrum: phytochemical characterization of five *Equisetum* species and evaluation of their antioxidant potential. Molecules **29**: 2821.

**Pallerla, P., Bhumireddy, S.R., Lingampally, S.S., Ragi, N.C., and Sripadi, P.** (2019). ESI-MS/MS analysis of protonated *N*-methyl amino acids and their immonium ions. J. Mass Spectrom. **54**: 761–771.

**Park, J.A., Kim, H.J., Jin, C., Lee, K.-T., and Lee, Y.S.** (2003). A new pterocarpan, (-)-maackiain sulfate, from the roots of *Sophora subprostrata*. Arch. Pharm. Res. **26**: 1009–1013.

**Pothier, J.F., Wisniewski-Dyé, F., Weiss-Gayet, M., Moënne-Loccoz, Y., and Prigent-Combaret, C.** (2007). Promoter-trap identification of wheat seed extract-induced genes in the plant-growth-promoting rhizobacterium *Azospirillum brasilense* Sp245. Microbiol. Read. Engl. **153**: 3608–3622.

**Qiao, X., Ye, M., Xiang, C., Wang, Q., Liu, C.-F., Miao, W.-J., and Guo, D.-A.** (2012). Analytical strategy to reveal the *in vivo* process of multi-component herbal medicine: a pharmacokinetic study of licorice using liquid chromatography coupled with triple quadrupole mass spectrometry. J. Chromatogr. A **1258**: 84–93.

**Saleh, N.A.M., Boulos, L., El-Negoumy, S.I., and Abdalla, M.F.** (1982). A comparative study of the flavonoids of *Medicago radiata* with other *Medicago* and related *Trigonella* species. Biochem. Syst. Ecol. **10**: 33–36.

**Singh, R., Wu, B., Tang, L., Liu, Z., and Hu, M.** (2010). Identification of the position of mono-O-glucuronide of flavones and flavonols by analyzing shift in online UV spectrum (lambda max) generated from an online diode array detector. J. Agric. Food Chem. **58**: 9384–9395.

**Staszków, A., Swarcewicz, B., Banasiak, J., Muth, D., Jasiński, M., and Stobiecki, M.** (2011). LC/MS profiling of flavonoid glycoconjugates isolated from hairy roots, suspension root cell cultures and seedling roots of *Medicago truncatula*. Metabolomics **7**: 604–613.

**Stochmal, A., Kowalska, I., Janda, B., Perrone, A., Piacente, S., and Oleszek, W.** (2009). Gentisic acid conjugates of *Medicago truncatula* roots. Phytochemistry **70**: 1272–1276.

**Stochmal, A., Piacente, S., Pizza, C., De Riccardis, F., Leitz, R., and Oleszek, W.** (2001). Alfalfa (*Medicago sativa* L.) flavonoids. 1. Apigenin and luteolin glycosides from aerial parts. J. Agric. Food Chem. **49**: 753–758.

**Szklarczyk, D. et al.** (2023). The STRING database in 2023: protein-protein association networks and functional enrichment analyses for any sequenced genome of interest. Nucleic Acids Res. **51**: D638–D646.

**Wen, X.-D., Qi, L.-W., Chen, J., Song, Y., Yi, L., Yang, X.-W., and Li, P.** (2007). Analysis of interaction property of bioactive components in Danggui Buxue Decoction with protein by microdialysis coupled with HPLC–DAD–MS. J. Chromatogr. B **852**: 598–604.

**Ye, X., Tang, M., Chen, L., Peng, A., Ma, L., and Ye, H.** (2009). Rapid separation and identification of major constituents in *Pseudolarix kaempferi* by ultra-performance liquid chromatography coupled with electrospray and quadrupole time-of-flight tandem mass spectrometry. Rapid Commun. Mass Spectrom. **23**: 3954–3962.

**Yoshikawa, K., Eiko, K., Mimura, N., Kondo, Y., and Arihara, S.** (1998). Hovetrichosides C−G, five new glycosides of two auronols, two neolignans, and a phenylpropanoid from the bark of *Hovenia trichocarea*. J. Nat. Prod. **61**: 786–790.

**Zhang, X., Xiao, H.-B., Xue, X.-Y., Sun, Y.-G., and Liang, X.-M.** (2007). Simultaneous characterization of isoflavonoids and astragalosides in two *Astragalus* species by high-performance liquid chromatography coupled with atmospheric pressure chemical ionization tandem mass spectrometry. J. Sep. Sci. **30**: 2059–2069.

**Zhang, Y., Nie, M., Shi, S., You, Q., Guo, J., and Liu, L.** (2014). Integration of magnetic solid phase fishing and off-line two-dimensional high-performance liquid chromatography–diode array detector–mass spectrometry for screening and identification of human serum albumin binders from Radix Astragali. Food Chem. **146**: 56–64.

**Zhou, L., Zhang, Q., Qi, W., Yan, S., Qu, J., Makino, T., and Yuan, D.** (2017). Identification of metabolites in human and rat urine after oral administration of Xiao-Qing-Long-Tang granule using ultra high-performance liquid chromatography combined with quadrupole time-of-flight mass spectrometry. J. Sep. Sci. **40**: 3582–3592.
